# Candidate Effectors of *Plasmodiophora brassicae* Pathotype 5X During Infection of Two *Brassica napus* Genotypes

**DOI:** 10.3389/fmicb.2021.742268

**Published:** 2021-11-03

**Authors:** Leonardo Galindo-González, Sheau-Fang Hwang, Stephen E. Strelkov

**Affiliations:** Department of Agricultural, Food & Nutritional Science, University of Alberta, Edmonton, AB, Canada

**Keywords:** clubroot, *Plasmodiophora brassicae*, effectors, pathotype 5X, *Brassica napus*

## Abstract

Clubroot, caused by *Plasmodiophora brassicae*, is one of the most important diseases of canola (*Brassica napus*) in Canada. Disease management relies heavily on planting clubroot resistant (CR) cultivars, but in recent years, new resistance-breaking pathotypes of *P. brassicae* have emerged. Current efforts against the disease are concentrated in developing host resistance using traditional genetic breeding, omics and molecular biology. However, because of its obligate biotrophic nature, limited resources have been dedicated to investigating molecular mechanisms of pathogenic infection. We previously performed a transcriptomic study with the cultivar resistance-breaking pathotype 5X on two *B. napus* hosts presenting contrasting resistance/susceptibility, where we evaluated the mechanisms of host response. Since cultivar-pathotype interactions are very specific, and pathotype 5X is one of the most relevant resistance-breaking pathotypes in Canada, in this study, we analyze the expression of genes encoding putative secreted proteins from this pathotype, predicted using a bioinformatics pipeline, protein modeling and orthologous comparisons with effectors from other pathosystems. While host responses were found to differ markedly in our previous study, many common effectors are found in the pathogen while infecting both hosts, and the gene response among biological pathogen replicates seems more consistent in the effectors associated with the susceptible interaction, especially at 21 days after inoculation. The predicted effectors indicate the predominance of proteins with interacting domains (e.g., ankyrin), and genes bearing kinase and NUDIX domains, but also proteins with protective action against reactive oxygen species from the host. Many of these genes confirm previous predictions from other clubroot studies. A benzoic acid/SA methyltransferase (*BSMT*), which methylates SA to render it inactive, showed high levels of expression in the interactions with both hosts. Interestingly, our data indicate that E3 ubiquitin proteasome elements are also potentially involved in pathogenesis. Finally, a gene with similarity to indole-3-acetaldehyde dehydrogenase is a promising candidate effector because of its involvement in indole acetic acid synthesis, since auxin is one of the major players in clubroot development.

## Introduction

Clubroot disease, which results from infection by the soilborne obligate parasite *Plasmodiophora brassicae* Wor., causes significant damage to plants in the Brassicaceae family, and specifically to crops of agricultural importance like canola (*Brassica napus* L.) (Dixon, 2009). While strategies for disease management including soil amendments, fungicides, and the sanitization of equipment hold some promise, the best approach for controlling clubroot has been the planting of resistant cultivars (Donald and Porter, 2009; Hwang et al., 2014). However, short rotations with clubroot resistant (CR) cultivars can result in resistance breakdown and the emergence of more virulent pathotypes (Cao et al., 2020). The emergence of new pathotypes of *P. brassicae* (Strelkov et al., 2016, 2018; Hollman et al., 2021) is therefore related to crop management practices and selection pressure on the pathogen, reflecting the arms race between the molecular defense mechanisms of the host and the generation of pathogen effectors utilized to overcome resistance.

*Plasmodiophora brassicae* is a resilient pathogen that produces resting spores that can remain in the soil for periods of up to 20 years (Hwang et al., 2012). The resting spores germinate to produce primary zoospores, which invade host root hairs and epidermal cells to generate primary plasmodia. These cleave into zoosporangia, from which secondary zoospores are released back into the soil. The secondary zoospores infect the root cortex, establishing a secondary infection through secondary plasmodia that will eventually give rise to a new generation of resting spores (Kageyama and Asano, 2009; Liu et al., 2020). Secondary plasmodia are responsible for the hypertrophy and hyperplasia of the host tissues through host hormone regulation (Jahn et al., 2013; Schuller et al., 2014; Ludwig-Müller et al., 2017). Increased root growth aids in housing the enlarged secondary plasmodia and provides a sink for nutrients utilized by the pathogen, and it is likely that most of the molecular interactions between host and pathogen take place during this stage, making this phase relevant in effector secretion and pathogenesis (Pérez-López et al., 2020, 2021).

Effectors from plant pathogens aid in the infection process either by directly affecting the immune response of the host, or by altering plant responses for their own benefit. These effectors are usually secreted proteins that can either act on the apoplast or enter plant cells, where they interact with different cellular components to cause disease and/or bind receptors (*R-*genes) or mediator proteins associated with R proteins that trigger immunity (Dodds and Rathjen, 2010; Toruño et al., 2016). Effectors can have diverse functions, including protection of the pathogen, protection of other effectors, renaturation of effectors that have been damaged by host reactive oxygen species (ROS), interference and/or manipulation of host gene expression and metabolism, manipulation of vesicular traffic or inhibition of pathogen-associated molecular patterns (PAMPs) (Toruño et al., 2016).

Effector proteins usually possess specific domains or motifs that allow their secretion. For example, an N-terminal signal peptide is typical of proteins that are processed through the secretory pathway. Different types of pathogens have distinct signatures depending on their infection characteristics. While oomycete effectors seem to have some typical signals in their N-terminal regions, this feature seems to be more variable in fungi (Selin et al., 2016). For example, intracellular effectors need translocation motifs like RxLR. In the case of *P. brassicae*, it has been speculated that motifs like RxLR, DEER and PEXEL are potentially necessary (Pérez-López et al., 2018), at least for some of the secreted proteins, to fulfill their function. It has been suggested, however, that the conservation of pathogenic effectors might reside in their tertiary and quaternary protein structures (Selin et al., 2016), making specific folds and structure more predictive of function since effectors tend to evolve rapidly at their primary sequence level (Mukhi et al., 2020). Additionally, many effectors are said to be small proteins (less than 300 or 400 amino acids), and bear a high proportion of cysteines, but not all effectors are necessarily small or rich in cysteines (Gout et al., 2006; Sperschneider et al., 2015).

So far, in *P. brassicae*, putative effectors have been identified through seminal studies on the *P. brassicae* genomes (Schwelm et al., 2015; Rolfe et al., 2016). Schwelm et al. (2015) predicted 553 secreted proteins in *P. brassicae* from genomic data, showing that these putative proteins were rich in ankyrin domains, which are essential for protein-protein interactions, and RING-domains, which can modify ubiquitination processing in the host. Other features common to many plant pathogens, like high proportions of cysteines, chitin recognition and protease inhibition, were common in the small secreted proteins of *P. brassicae*, but a general enrichment of translocation motifs like RxLR was not evident. In the meantime, Rolfe et al. (2016) predicted 590 secreted proteins, 221 of which were classified as small-secreted proteins (<300 aa based on their classification). This study also confirmed a low prevalence of the RxLR translocation motif, with only 13 proteins showing the motif. Additionally, differential expression of the effectors, 6 weeks after inoculation, showed that genes corresponding to these proteins were likely interfering with the host SA-induced defenses, most likely through the action of benzoic acid/salicylic acid methyltransferase (Pb3-BSMT) (Rolfe et al., 2016). *PbBSMT* was shown to be one of the most highly expressed genes in galled tissue of *Brassica oleracea* (Ciaghi et al., 2019). Functional analysis to examine the putative effect of this *P. brassicae* effector on host plants indicated that it could interfere with host induced SA-triggered defenses (Ludwig-Müller et al., 2015), and localization analyses have shown the appearance of *BSMT* mRNA during early secondary infection and early sporogenesis (Badstöber et al., 2020). Furthermore, overexpression of *PbBSMT* in host Arabidopsis plants caused a reduction in SA production and higher susceptibility to clubroot (Bulman et al., 2019; Djavaheri et al., 2019).

A pipeline was proposed to discover effector proteins based on putative secretion and typical motifs needed for translocation into host cells (Pérez-López et al., 2018). This pipeline was then used to predict 32 small-secreted proteins (SSPs) of <400 aa that were rich in cysteine (Pérez-López et al., 2018). Few of the predicted proteins had the expected translocation motifs (RxLRR and PEXEL), with some proteins bearing ankyrin domains. Kinase functional activity was confirmed for one of the predicted SSPs.

Effectors were traditionally identified using genetic map-based cloning (Selin et al., 2016), but with the advent of Next Generation Sequencing (NGS) technologies, studies in genomics and transcriptomics provide the raw data for prediction of putative effector candidates expressed during the infection cycle. Here we have implemented a pipeline for traditional and non-traditional effector prediction in a *P. brassicae* pathotype 5X infecting two *B. napus* genotypes that previously showed divergent molecular responses to this pathotype (Galindo-González et al., 2020). Pathotype 5X, as classified on the Canadian Clubroot Differential (CCD) set (Strelkov et al., 2018), was one of the first pathotypes to break first generation resistance in clubroot-resistant (CR) canola cultivars (Strelkov et al., 2016). Many of the proteins and domains previously predicted as *P. brassicae* effectors in other studies (Ludwig-Müller et al., 2015; Schwelm et al., 2015; Yahaya et al., 2015; Pérez-López et al., 2020) were identified in the current study, but some novel candidates are also outlined. Structural modeling of the proteins helped in predicting effector functions in some cases where comparison of the primary amino acid sequence did not result in a putative function.

While traditionally the management of plant pathogens has centered on developing host resistance, the study of pathogen infection mechanisms can help to pinpoint potential interactions that can be targeted to improve plant resistance or to interfere with pathogen compatibility. These interactions are being shown to be quite specific between pathotypes and host genotypes, and therefore, an understanding of both the specific mechanisms of virulence by each pathogen and the common mechanisms of virulence may help in the development of effective host resistance.

## Materials and Methods

### Experimental Set Up and Data Analysis

Data analyzed in the current study comes from previously published research where host molecular responses were assessed (Galindo-González et al., 2020). The materials and experiments performed previously are briefly described below for context, and novel analyses performed for the current study on the raw reads corresponding to the pathogen are described in detail.

### Pathogen Material and Host Inoculation

Experiments corresponding to the current study have been previously performed and published (Galindo-González et al., 2020). Briefly, inoculum of *P. brassicae* pathotype 5X, as defined on the Canadian Clubroot Differential (CCD) set (Strelkov et al., 2018), was adjusted to a concentration of 1 × 10^7^ resting spores/mL with sterile distilled water. Two *B. napus* genotypes, the oilseed rape ‘Brutor’ and rutabaga ‘Laurentian,’ susceptible and partially resistant to pathotype 5X, respectively, were inoculated by the root-dip method and grown in parallel with non-inoculated controls; the roots were harvested at 7, 14, and 21 days after inoculation (dai) for each of the control vs. inoculated treatments and both host genotypes.

### RNA Extraction and RNAseq

RNA is also from a previous published experiment (Galindo-González et al., 2020), where only the host fraction was analyzed (Galindo-González et al., 2020). Briefly, RNA was extracted from frozen roots of both host genotypes, and RNAseq was performed from 3 μg of RNA per sample with RNA Integrity numbers >9, as previously described (Galindo-González et al., 2020). Sequencing was conducted at Oklahoma State Genomics (Stillwater, OK, United States).

For the current study, after mapping reads to the host *B. napus* reference genome (Genbank assembly: AST_PRJEB5043_v1) using TopHat, the unmapped bam files were sorted and transformed into fastq files using samtools v1.10 (Li et al., 2009) and bedtools v2.27.1 (Quinlan and Hall, 2010). Reads were checked for quality using fastqc v0.11.8^1^, and filtered for quality and adapter presence using a minimum per base phred quality value of 30, a minimum read length of 70 and polyG and polyX trimming with a minimum length of 10 bases; the rest of the parameters were left by default.

Coding sequences (CDS’) from the e3 *P. brassicae* genome (Stjelja et al., 2019) were downloaded from the European Nucleotide Archive (WGS project OVEO01000000), and the filtered reads were mapped and quantified to all 9284 CDS’ with Salmon v1.1.0 (Roberts and Pachter, 2013) using an indexed transcriptome, mapping validation, a mean read size of 75 bases, a mean read size standard deviation of 5, 50 bootstrap repetitions and GC bias correction. Differential expression analysis of mapped reads was performed using Deseq2 (Love et al., 2014) by comparing the *P. brassicae* quantified reads from both genotypes at each time-point. Significant differential gene expression was assessed by comparing the TPM (Transcripts Per kilobase Million) dispersion (Deseq2 dispersion based on mean and variance) of three replicates with adjusted *p-*values (after multiple comparisons) that were equal or below 0.05. Putative secreted proteins were selected with the pipeline described in the section below for genes having at least 10 mapped reads in at least one of the two samples being compared. Additionally, we performed an analysis of these genes for each time-point and sample (*P. brassicae* reads coming from infection of ‘Laurentian’ or ‘Brutor’) for genes which showed <20% covariance in their TPMs, and at least 10 mapped reads on average across three biological replicates.

### Secretome Bioinformatics Pipeline

Coding sequences were first annotated by comparing them to the non-redundant (nr) protein Genbank database (downloaded 2020-04-22) and to the Uniprot database (downloaded 2020-08-31), using BLASTx (Altschul et al., 1990) with a minimum e-value of 1e-10. Gene annotation from the comparison against nr was performed by submitting the resulting xml file from BLASTx to BLAST2GO (Conesa et al., 2005; Conesa and Götz, 2008), which performs a consensus annotation from the top 20 hits of each CDS. Uniprot top hit IDs for each CDS were searched on ProSecKB (Powell et al., 2016), a database for protist secretomes, using *P. brassicae* to search subcellular localization. Pfam and Interpro domain predictions were performed with InterproScan (Quevillon et al., 2005; Hunter et al., 2009).

Putative translations of CDS’ were used for secretome prediction. SignalP v4.1f (Nielsen, 2017) was used to predict a signal peptide (presence of signal peptide = Y) and potential cleavage sites, and SecretomeP v1.0h (Bendtsen et al., 2004) was used to predict non-traditional leaderless secreted proteins with a minimum score of 0.5. Proteins with a valid prediction from these two tools were filtered further using TargetP v2.0 (Armenteros et al., 2019), selecting proteins predicted to be targeted for the secretory pathway or unknown pathway “OTHER,” while the proteins carrying chloroplast or mitochondria transit peptides were discarded. The software TMHMM v2.0 (Sonnhammer et al., 1998) was then used for transmembrane helix prediction, where proteins with two or more helices or with one helix non-overlapping a predicted signal peptide were filtered out. From the remaining sequences, GPI-anchored proteins were discarded using PredGPI (Pierleoni et al., 2008) and NetGPI v1.0 (Gíslason et al., 2021). Extracellular proteins were predicted with WolfPsort (Horton et al., 2007) and Deeploc v1.0 (Almagro Armenteros et al., 2017). These proteins were depicted as secreted and were further analyzed with EffectorP v2.0 (Sperschneider et al., 2018a) and ApoplastP (Sperschneider et al., 2018b). Motifs typically found on effectors (RxLR, Pexel and DEER) (Pérez-López et al., 2018) were searched with Fuzzpro^2^. The percentage of cysteines was calculated, and the protein was classified as cysteine-rich if this percentage was equal or larger than 2.5%. A protein was classified as a small-secreted protein (SSP) if its size was <400 amino acids (Pérez-López et al., 2018).

Our pipeline is similar to the one used by Pérez-López et al. (2018), but uses additional tools for prediction of non-conventional secretion (SecretomeP), membrane anchors (PredGPI and NetGPI), and a dual approach for determining extracellular secretion (WolfPsort + Deeploc). Additionally, we added EffectorP, a tool to try to distinguish between proteins that are secreted and those that exclusively characterized as effectors. Finally, our search for the RxLR, Pexel, and DEER motifs was done automatically with Fuzzpro, as opposed to the manual search performed in the previous studies (Pérez-López et al., 2018, 2020).

### Orthologous Proteins With Virulence Changes

All non-redundant differentially expressed transcripts and low covariance transcripts were putatively translated and submitted for a PHIB-BLAST at the pathogen–host interactions database (PHI-base) (Urban et al., 2020) using the default parameters. This comparison enabled matching of the predicted *P. brassicae* secreted proteins with closely related proteins from other pathogens that have been functionally tested for their effects on hosts. Hits with e-values below 1e-5 and showing a change in virulence upon experimental gene alteration were selected for the analysis.

### Protein Structural Prediction

Protein structural prediction was performed with Swiss-Model (Waterhouse et al., 2018) under automated settings. The list of potential templates was checked to confirm that the resulting model was generated from the best template according to sequence alignment and coverage, and to the generated model as assessed by the Global Model Quality Estimation (GMQE) score^3^. Protein structural alignment was performed with PyMol v2.4 (The PyMOL Molecular Graphics System, Version 2.4 Schrödinger, LLC.) with a one-to-one comparison, default alignment, outlier rejection for 5 cycles, a cut-off of 2.0 and mobile and target states of −1. When alignment presented high RMSDs, they were aligned with the super and cealign modes, which work when there is low primary sequence similarity but some structural conservation.

### Validation of Expression Levels From Transcripts Corresponding to Secreted Proteins

Validation was performed for 28 target genes predicted as secreted and five housekeeping genes (Supplementary Table 1) using a nanostrings nCounter^®^ Custom Codeset (Nanostrings, Seattle, WA, United States). Target and reporter probes were designed by Nanostrings using the sequences from the latest e3 *P. brassicae* genome (Stjelja et al., 2019). Probe design by Nanostrings is based on proprietary algorithms that interrogate target sequences in windows of 100 nucleotides shifting one nucleotide at a time. Probes were screened for hybridization efficiency, cross hybridization (no more than 85% sequence homology between two sequences and no more than 16 consecutive matches), GC content and secondary structure (Nanostrings Technologies, 2011). One microgram of RNA per sample was hybridized to probes using a standard nanostrings hybridization protocol (Nanostrings Technologies, 2021b) and run on a nCounter^®^ SPRINT Profiler (Nanostrings, Seattle, WA, United States). Analysis of data was performed using the nSolver Analysis Software 4.0 (Nanostrings, Seattle, WA, United States). Normalized log_2_ ratio data from differentially expressed genes and log_2_ transformed normalized count values were used for comparison to differentially expressed genes and transcripts with low covariance among biological replicates in the RNAseq data.

### BLAST Analysis of Candidate Effectors

The most relevant putative secreted transcript sequences were used to perform a BLASTn search against the genome assemblies of isolates Pb3 (Rolfe et al., 2016), eH (Daval et al., 2019), and ZJ-1 (Bi et al., 2019). Genomes from the three isolates were downloaded from Genbank^4^, and formatted as a blastable database using makeblastd. The query sequences were blasted against the three databases using a minimum *e*-value of 1e-10.

## Results and Discussion

### Differentially Expressed Secretome

There were no differentially expressed genes at 7 dai when comparing *P. brassicae* transcripts coming from infection of the resistant host genotype ‘Laurentian’ vs. the susceptible genotype ‘Brutor.’ This could be due to a lower number of pathogenic cells at the beginning of secondary infection, which was demonstrated by lack of symptoms in the both host genotypes at this time point (Galindo-González et al., 2020). At 14 dai, only two genes were differentially expressed and just one was predicted to be a secreted protein (SPQ95221.1) in our pipeline. This *P. brassicae* transcript was downregulated in the roots of the resistant genotype ‘Laurentian’ compared with the roots of ‘Brutor’ (log_2_FC = −0.36) and was annotated as a hypothetical protein with no recognized domains. The predicted protein did not have a signal peptide, but was classified by SecretomeP as a non-traditional secreted protein, which was supported by the ProSecKB classification. Interestingly, the transcript corresponding to this protein also showed the highest relative abundance (TPM) from the differentially expressed secreted genes (DESGs) at 21 dai in *P. brassicae* cells from the two host genotypes, but with an opposite trend with the gene being more abundant in *P. brassicae* infecting ‘Laurentian.’ The transcript levels of SPQ95221.1, as calculated by TPM, decreased in *P. brassicae* infecting ‘Laurentian’ from 29758.99 at 14 dai to 26366.57 at 21 dai. In ‘Brutor’ the decrease was more substantial, with a TPM of 37719.81 at 14 dai and of 11681.39 at 21 dai.

At 21 dai, 21 differentially expressed transcripts were identified as secreted proteins, with only nine being SSPs based on their size (Table 1). These transcripts had higher TPMs in *P. brassicae* infecting ‘Laurentian.’ Pfam annotation of the 21 putatively secreted proteins showed that nine possessed ankyrin domains and two could be classified as SSPs (SPQ95835.1 and SPQ95830.1). These two proteins were also identified by Pérez-López et al. (2020) as small secreted proteins (SSPbP43 and SSPbP93), following challenge of Arabidopsis with *P. brassicae*. Transcripts corresponding to genes SPR01793.1 and SPR01853.1 (Table 1) in our study were also identified in the Arabidopsis study as SSPbP31 and SSPbP42, but did not have ankyrin domains. Ankyrin domain repeats mediate protein-protein interactions, which are essential for the effector-receptor relationship and have been shown to be involved in cell cycle, cell proliferation of host cells and, potentially, *P. brassicae* life cycle transitions (Pérez-López et al., 2020). Interestingly, EffectorP and ApoplastP, which are machine-learning methods, did not predict any of the ankyrin bearing protein sequences as either being effectors or apoplastic. In fact, only three of the 21 proteins were classified as effectors by EffectorP (SPQ99629.1, SPQ98385.1, and SPR01853.1). Another three had both an RxLR and a Pexel motif (SPR00206.1, SPR00984.1, and SPQ99289.1); these domains are relevant in translocation and secretion (Wawra et al., 2013, 2017; Pérez-López et al., 2020). SPR00984.1 also presented one of the highest levels of expression from our analysis.

**TABLE 1:** Secretome prediction of differentially expressed *P. brassicae* transcripts 21 dai.

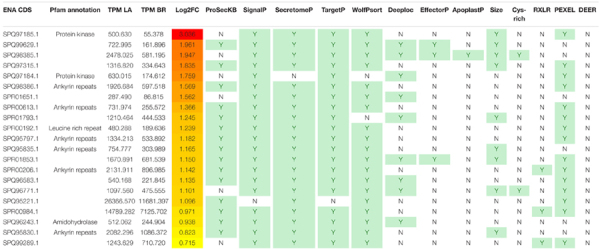

*ENA CDS, European nucleotide archive accession no.*

*Pfam, protein domain.*

*TPM, average from 3 biological replicates for expression level of *P. brassicae* transcripts coming from the interaction with ‘Laurentian’ (LA) or ‘Brutor’ (BR).*

*Log2FC, fold change heatmap of the relationship between *P. brassicae* transcript expression level in hosts ‘Laurentian’ and ‘Brutor. Minimum = 0.71 (yellow), Maximum = 3.03 (red).*

*For each tool, shaded cells (Y) mean presence.*

*ProSecKB, characterized as secreted.*

*SignalP, signal peptide present.*

*SecretomeP, >0.5 score.*

*TargetP, secretory pathway.*

*WolfPsort/Deeploc, extracellular localization.*

*EffectorP, effector.*

*ApoplastP, secreted to apoplast.*

*Small proteins, equal or <400 aa.*

*Cyst-rich, equal or >2.5%.*

*RXLR/PEXEL/DEER, presence of motif.*

Two DESGs (SPQ97184.1 and SPQ97185.1) encoded proteins with kinase domains, a domain which has been reported in studies of secreted proteins of *P. brassicae* (Schwelm et al., 2015; Pérez-López et al., 2020). SPQ97185.1 showed the highest differential expression of any transcript at 21 dai (Table 1) and can be classified as a SSP. Finally, another protein (SPR00192.1) had a leucine-rich repeat (LRR) domain; this domain was also reported as enriched in secreted proteins (Schwelm et al., 2015).

The second gene with the highest differential expression (SPQ99629.1) did not have any Pfam domains, and was annotated as a 30S ribosomal subunit 19 (Supplementary Table 2). Seven of eight tools predicted this gene for extracellular secretion. Although it might seem counterintuitive for a ribosomal subunit to be secreted, ribosomal proteins are part of the secretome of *Trichoderma virens* during its interaction with maize roots (Nogueira-Lopez et al., 2018). Furthermore, knocking down a 40S ribosomal subunit from *Puccinia striiformis* f. sp. *tritici* (*Pst*) resulted in a decreased pathogenicity on infected wheat (Wang et al., 2019). While the protein from *Pst* is not secreted, ribosomal proteins from pathogens could likely be involved in numerous plant-pathogen interactions.

**FIGURE 1:**
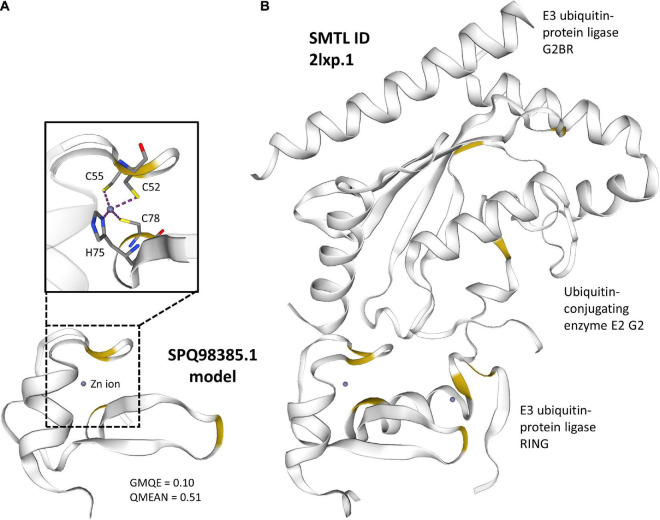
Predicted 3D structure of E3 Ubiquitin-Protein Ligase RING subunit. **(A)** The region corresponding to the RING region of the E3 ubiquitin protein ligase was predicted using Swiss-model. A Global Model Quality Estimation (GMQE) score closer to 1 represents more accuracy in the models, and a QMEAN value above –4 is indicative of good quality of the structure (https://swissmodel.expasy.org/docs/help). The zinc-binding region is indicated by a dashed square on the model with the metal complexes between Zn and amino acids shown above. **(B)** The selected Swiss-Model Template (SMTL) to create the model of the *P. brassicae* protein is a complex of three units including two E3 ligases and an ubiquitin-conjugating E2 enzymes. Cysteine regions in both models are highlighted in yellow.

**FIGURE 2:**
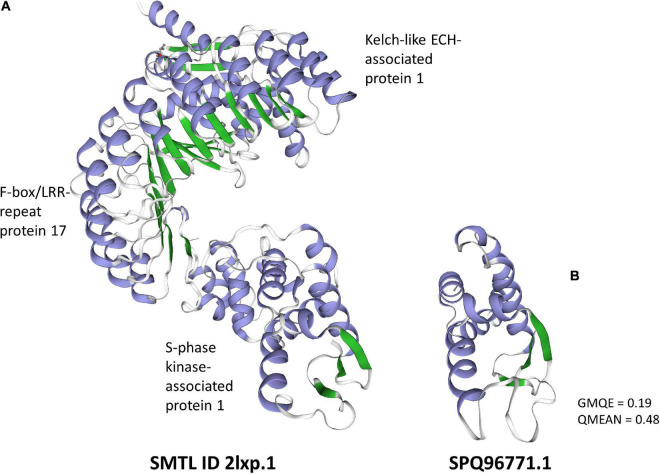
Predicted 3D structure of S-phase kinase-associated protein 1. **(A)** The model or the heterotrimeric complex used as template. **(B)** The region corresponding to the S-phase kinase-associated protein 1 was predicted using Swiss-model. A Global Model Quality Estimation (GMQE) score closer to 1 represents more accuracy in the models, and a QMEAN value above –4 is indicative of good quality of the structure (https://swissmodel.expasy.org/docs/help).

The third gene with the highest differential expression (SPQ98385.1) did not have a blast match or a recognizable protein domain. However, most of the tools predict this gene as secreted and it is the only sequence in this list classified as both an effector by EffectorP and as secreted to the apoplast by ApoplastP (Table 1). The predicted protein is also the right size for a SSP and has a high cysteine content (5.1% – Supplementary Table 2). This gene constitutes an important candidate for functional analysis, and therefore we built a 3D model using Swiss model to discover the potential function of the encoded protein. The predicted structure was similar to the E3 ubiquitin-protein ligase RING subunit (Figure 1A) of the ubiquitin ligase gp78, which forms a heterotrimeric complex with another protein ubiquitin ligase and an ubiquitin-conjugating E2 enzyme (Figure 1B). The glycoprotein Gp78 is an E3 ubiquitin ligase that prevents accumulation of misfolded proteins (Joshi et al., 2017), but also seems to be involved in cell proliferation in different cancerous cells. E3 ubiquitin ligases are involved in protein modulation and more often in protein degradation via the proteasome. Characterization of E3 ubiquitin ligases from the *P. brassicae* genome indicated that 11 RING-type E3 ligases had a signal peptide and were likely to be secreted (Yu et al., 2019). While an effector function has not been validated for these proteins, it has been speculated they could aid in targeting host receptors for degradation, therefore favoring susceptibility of the host (Yu et al., 2019). Interestingly, the structural model of another one of our predicted *P. brassicae* secreted proteins (SPQ96771.1 – Figure 2) with similar characteristics to SPQ98385.1, but without positive predictions on EffectorP or ApoplastP, was similar to an S-phase kinase-associated protein 1 (SKP1 – Swiss model ID: 6w66.1, GMQE = 0.16, QMEAN, –2.66). This protein is an important component of the SCF complex (Skip1-Cullin1-F-box) E3 ubiquitin ligase complex that is formed for protein degradation through the 26S proteasome. The heterotrimeric complex used as template for modeling SPQ96771.1 is involved in ubiquitination and degradation of inactive heterodimers of BTB proteins (Mena et al., 2020). | Mutational analysis of the blast rice fungus Skp1 has shown that this gene is essential for fungal development and pathogenicity (Prakash et al., 2016), indicating how proteins involved in protein degradation may be important candidates for pathogenicity in *P. brassicae*.

### Consistently Expressed Genes

**FIGURE 3:**
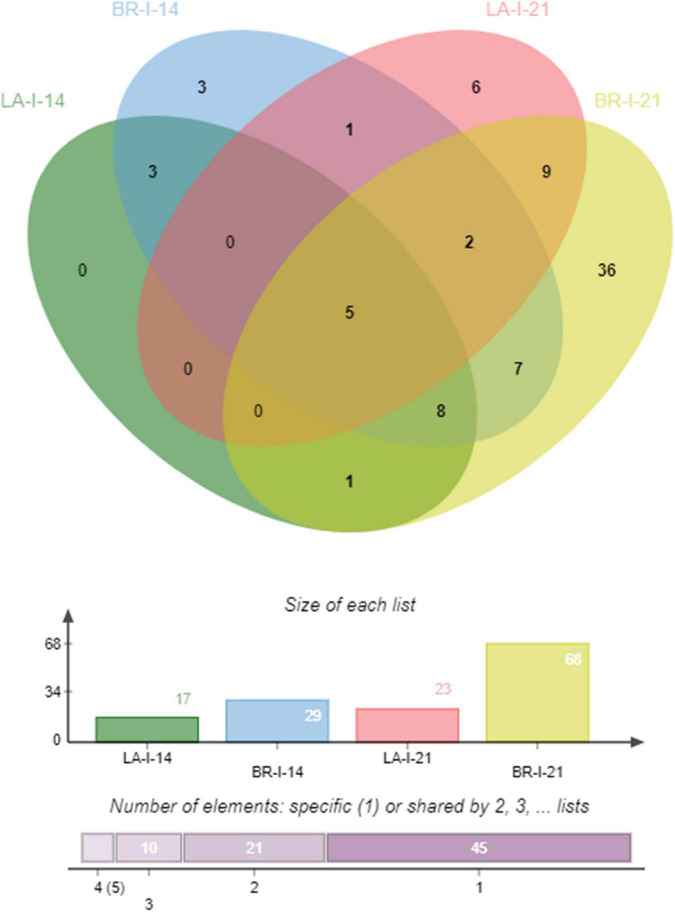
Venn diagram of consistently regulated transcripts corresponding to secreted proteins. The Venn diagram depicts the number of *P. brassicae* transcripts with common, opposite and distinct expression patterns coming from the two *B. napus* hosts at 14 and 21 dai. LA-I-21 = ‘Laurentian’ inoculated 21 dai; BR-I-21 = ‘Brutor’ inoculated 21 dai.

To identify genes that showed consistent high expression (low variability among biological replicates), and that can uncover effectors affecting both hosts, we looked at the *P. brassicae* transcripts within each genotype and time-point having <20% covariance in their TPM across the three biological replicates per treatment. We found no *P. brassicae* transcripts with low covariance among biological replicates at 7 dai in either genotype, while 17 and 29 transcripts corresponding to secreted proteins under our pipeline showed low covariance at 14 dai in *P. brassicae* cells from ‘Laurentian’ and ‘Brutor,’ respectively. At 21 dai, these numbers increased to 23 *P. brassicae* transcripts from the interaction with ‘Laurentian’ and 68 with ‘Brutor’ (Figure 3).

#### Shared *P. brassicae* Transcripts at 14 and 21 Dai From Both Hosts

Five transcripts showed consistent expression in *P. brassicae* infecting both host genotypes at 14 and 21 dai (Figure 3 and Supplementary Tables 3–5). Only one of these five transcripts (SPQ99289.1) was differentially expressed in *P. brassicae* samples coming from the two hosts at 21 dai (Supplementary Table 5).

The transcript with the highest expression across both days and genotypes was a SAM (*S*-adenosyl-L-methionine)-dependent carboxyl methyltransferase (SPQ93076.1 – Supplementary Table 5), also known as Benzoic acid/salicylic acid methyltransferase (BSMT). A higher TPM was evident for this transcript at 14 dai in *P. brassicae* infecting ‘Brutor,’ but the relationship was inverted at 21 dai (Supplementary Table 5). This is consistent with the increased expression of this gene between 2 and 4 weeks when *P. brassicae* colonized *B. napus* (Rolfe et al., 2016), and its presence during secondary infection using localization assays (Badstöber et al., 2020). Salicylic acid is usually inactive in its methylated form, which impairs SA-mediated defense responses. In our previous study, where we characterized the host responses of genotypes infected by pathotype 5X from the current study, SA-mediated responses were the main defense mechanism from hosts, with the more resistant cultivar displaying a stronger SA-mediated molecular defense (Galindo-González et al., 2020). Multiple studies in *B. napus, Brassica rapa* and Arabidopsis showed that SA and SA-mediated molecular responses increased in resistant cultivars when challenged with *P. brassicae*, and that the application of SA decreased clubroot symptom severity (Lemarie et al., 2015; Chen et al., 2016; Prerostova et al., 2018). The benzoic acid/salicylic acid methyltransferase from *P. brassicae* was shown to methylate salicylic acid (SA) in *in vitro* assays and its highest transcription level matches a high production of SA by the host (Ludwig-Müller et al., 2015). This gene also showed high levels of expression in gall tissue of *B. oleracea* when compared with asymptomatic root tissue in the same plants (Ciaghi et al., 2019), and its overexpression in host plants causes increased susceptibility and decreases in active SA levels (Bulman et al., 2019; Djavaheri et al., 2019). Transcripts of *PbBSMT* increase as clubroot disease progresses in Arabidopsis, and reads related to this gene were overrepresented in a callus culture of *B. rapa* infected with *P. brassicae* (Ludwig-Müller et al., 2015). The authors argued that increased expression of *PbBSMT* during secondary infection of *P. brassicae* in Arabidopsis would potentially be necessary to decrease the SA-mediated response of the host. This gene is also one of 32 SSPs previously predicted in the Arabidopsis-clubroot interaction (Pérez-López et al., 2020). From our bioinformatics analysis, BSMT is a SSP with RxLR (aa 211–214) and Pexel (aa 191–195) motifs. The protein sequence, however, was not predicted to be an effector or apoplastic by EffectorP or ApoplastP (Supplementary Table 5), and its percentage of cysteine (1.3%) was below our threshold for a cysteine-rich protein. Nevertheless, BSMT is probably one of the most important proteins from the *P. brassicae* secretome.

The second transcript (SPR00473.1) with highest expression among these five genes expressed from *P. brassicae* cells from both hosts at 14 and 21 dai was annotated as a luminal-binding protein 5/heat shock protein 70 (*Hsp70*) (Supplementary Table 5). Heat shock proteins aid in correct protein folding and are usually expressed under stress conditions where proteins tend to denature; although counterintuitive, the infection of the host by *P. brassicae* also exerts physiological stress upon the pathogen proteins which are directly secreted in the host cytoplasm. A heat shock protein 70 was one of 76 genes identified using subtractive hybridization in *P. brassicae*-infected Arabidopsis (Bulman et al., 2006). Heat shock protein transcript expression was detected in *P. brassicae* infecting Arabidopsis at 14, 21, and 28 dai (Siemens et al., 2009), and throughout a time-course from 6 to 41 dai in *B. rapa* (Wu et al., 2012). Interestingly, HSPs including HSP70 from *Plasmodium falciparum* have been implicated in inter-organelle trafficking and the export of proteins of parasitic origin (Shonhai, 2010). In fact, because of their nature, HSPs have been proposed as targets for antimalarial drugs that can inhibit HSP association with co-chaperons and their trafficking function (Shonhai, 2010; Kumar et al., 2018). Although a human parasite, there may be some similarities between pathogenesis involving *P. falciparum* and *P. brassicae* (Pérez-López et al., 2020). For example, the Pexel motif found in *P. falciparum* was suggested as a very likely motif directing translocation of effectors in *P. brassicae* (Marti et al., 2004; Pérez-López et al., 2020).

The third gene with highest transcript level (SPQ96353.1) was annotated as a peptide methionine sulfoxide reductase (*Msr*). This enzyme catalyzes the reduction of oxidized methionine in proteins back to methionine (Weissbach et al., 2002). Oxidation of sulfur groups in sulfur peptides like methionine is a common occurrence under highly oxidative environments, and would be favored when host cells enhance reactive oxygen species (ROS) production as part of their defense response. In one of our recent studies, three respiratory burst oxidase (RBOH) genes were upregulated in a resistant *B. napus* host in response to *P. brassicae* pathotype 3A (Zhou et al., 2020); similar genes were activated in resistant wild cabbage when challenged with this pathogen (Zhang et al., 2016). A peptide methionine sulfoxide reductase A (MsrA) from the bacterium *Erwinia chrysanthemi* was shown to be necessary for virulence on its plant hosts, since mutants of this gene resulted in lower virulence and no systemic invasion of the pathogen (El Hassouni et al., 1999). This demonstrates that host plants respond to the pathogen by activating ROS and that the pathogen activates antioxidant enzymes to survive in the oxidative environment. Furthermore, it is possible that antioxidant pathogen enzymes can protect the virulence factors by reversing their oxidation to allow disease progression (El Hassouni et al., 1999). The action of Msr proteins as important determinants of virulence is supported by studies of human pathogens, which have shown decreases in virulence when these genes are mutated (Alamuri and Maier, 2004; Denkel et al., 2011; Singh et al., 2018). Our characterization of SPQ96353.1 indicates that this gene encodes a cysteine-rich non-conventional SSP without a signal peptide. Additionally, the translated product was predicted to be an effector and apoplastic by EffectorP and ApoplastP, further suggesting a role in infection.

The last of these consistently expressed genes was a serine carboxypeptidase (SPR01261.1). A serine protease cloned from *P. brassicae* (PRO1) showed catalytic activity in a heterologous system and was upregulated in canola roots from 4 to 42 dai (Feng et al., 2010). This gene was proposed to be involved in resting spore germination. Peptidase/proteases were reported among predicted SSPs in seminal studies of the *P. brassicae* genome (Schwelm et al., 2015; Rolfe et al., 2016). In other plant hosts, serine carboxypeptidases have also been reported as likely virulence factors (Kim et al., 2004; Williams and Sternthal, 2010; Dalman et al., 2013).

#### *Plasmodiophora brassicae* Secretome Transcripts When Infecting ‘Brutor’ at 21 Dai

The majority of transcripts with low covariance came from *P. brassicae* infecting ‘Brutor’ at 21 dai (Supplementary Table 3). From the 68 transcripts, 36 were unique to the interaction with the host ‘Brutor’ at 21 dai (Bin2, Supplementary Table 3), while nine were shared exclusively with transcripts coming from inoculated ‘Laurentian’ plants at 21 dai (Bin 3, Supplementary Table 3), and 7 were shared exclusively with transcripts from ‘Brutor’ at 14 dai (Bin5, Supplementary Table 3). From all transcripts analyzed, a larger proportion of pathogen genes had higher expression when infecting ‘Brutor’ at 14 dai, while at 21 dai, more pathogen transcripts interacting with ‘Laurentian’ had a higher average TPM. However, the level of expression of the *P. brassicae* transcripts from the ‘Laurentian’ interaction at 21 dai presented high covariance, which resulted in these genes being identified mainly in *P. brassicae* coming from ‘Brutor’ (Supplementary Table 6).

From the 36 transcripts with low covariance identified from *P. brassicae* in infected ‘Brutor’ plants at 21 dai (Bin 2, Supplementary Table 3), the most common domain according to the Pfam annotation corresponded to Ankyrin repeats, as described in our differential expression analysis above. In addition to this domain, other domains identified indicated the importance of pathogen protection, detoxification and neutralization of oxidative damage, including 2OG-Fe (II) oxygenase, glutathione peroxidase, aldehyde dehydrogenase, thioredoxin, and short chain dehydrogenase. The 2OG-Fe (II) oxygenase (SPQ94350.1 – Supplementary Table 6), presented low covariance at 21 dai but was also expressed at 14 dai. This exact same gene [corresponding to PBRA003835 in the first genome draft of *P. brassicae* (Schwelm et al., 2015)] has been recently identified as significant inhibitor of programmed cell death (PCD) in the clubroot interaction, and designated as PbPE15 (Hossain et al., 2021). Another group of genes was annotated to processes of nutrient acquisition and transformation, including carbohydrates and nitrogen derivates metabolism (glucosidase II beta subunit, aldolase 1-epimerase, allantoicase, and amidohydrolase), which is in agreement with overall patterns of secretomes in biotrophic fungi (Kim et al., 2016).

**FIGURE 4:**
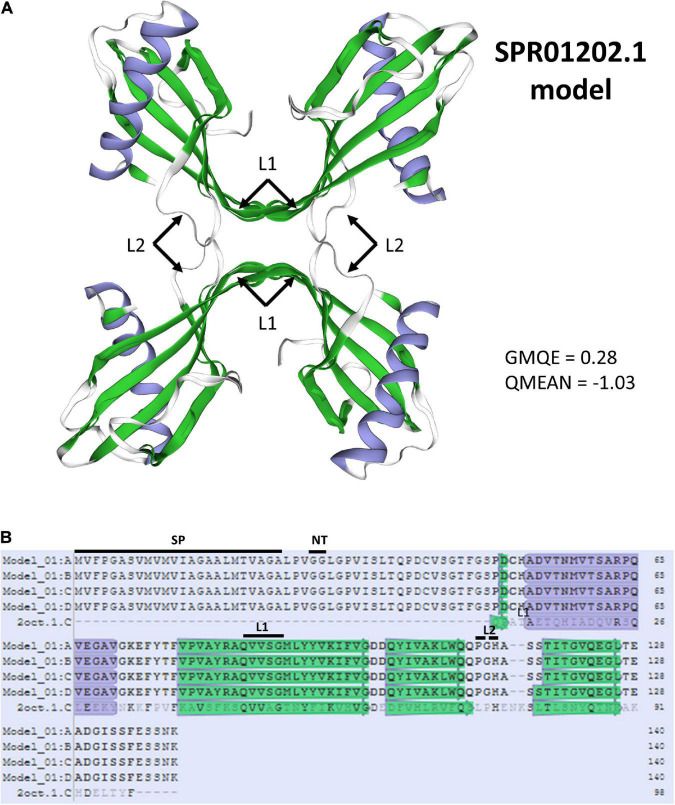
Predicted 3D structure of a Cystatin Protease Inhibitor. **(A)** The cystatin homo-tetramer was predicted using Stefin-B (Cystatin-B, STML ID:2oct.1.C) using Swiss-model. A Global Model Quality Estimation (GMQE) score closer to 1 represents more accuracy in the models, and a QMEAN value above –4 is indicative of good quality of the structure (https://swissmodel.expasy.org/docs/help). Alfa helices are depicted in purple and beta sheets and loop regions are in green; the four L1 and the four L2 loop regions are indicated. **(B)** Multiple sequence alignment of the four predicted monomers forming the tetramer with the template Stefin-B. Alpha helices are highlighted in purple and beta sheets are in green. The three conserved sites that form a complex with papain: the N-terminal Trunk (NT), loop 1 (L1), and loop 2 (2), are also shown.

Three previously reported SSPs (SSPbP31, SSPbP53, and SSPbP21)(Pérez-López et al., 2020) were among these 36 predicted proteins at 21 dai, and corresponded to genes SPR01793.1, SPR01202.1, and SPQ93225.1 in the current study. The three transcripts did not show similarity to any pfam domains in our study. Pérez-López et al. (2020) reported SSPbP53 as a cysteine protease inhibitor directed to the apoplast, consistent with the apoplastic prediction we obtained with ApoplastP (Supplementary Table 3). Furthermore, homology modeling of the protein (Figure 4) showed its similarity to a human Stefin B protein tetramer. Stefin B is a protease inhibitor from the cystatin family, which helps to protect the cells from protein misfolding and aggregation (Žerovnik, 2019), thereby reducing the impact of ROS. Protease inhibitors are important weapons in the virulence of fungi and oomycetes, by directly interacting with plant proteases and thus suppressing their activity (Wang et al., 2020). Further functional characterization of SSPbP53 has confirmed its function as a papain-like cysteine protease inhibitor in cruciferous plants (Pérez-López et al., 2021). Also, a cystatin-like protease inhibitor from the oomycete *Phytophthora palmivora* inhibits the activity of the papaya cysteine protease papain (Gumtow et al., 2018). Each of the cystatin monomers of the cystatin tetramer has a typical structure with an alpha helix on top of an antiparallel beta sheet as can be seen from the predicted model (Figure 4A). The monomers in the model contain two of the three predicted conserved sites (Nagata et al., 2000; Gumtow et al., 2018), a first binding loop (L1) made of a conserved QxVxG motif, and a second binding loop (L2), which usually contains a proline followed by an aromatic residue (H or W), although this latter loop seems interrupted by a Glycine (G) in the *P. brassicae* protein (Figure 4B). The third site could not be predicted on the 3D model, but corresponds to an N-terminal trunk (NT) that can be seen in the sequence from the multiple alignment (Figure 4B). These conserved sites form a hydrophobic wedge that tightly binds to the active cleft of papain (Bode et al., 1988; Turk and Bode, 1991). Finally, the last of these three SSPs (SSPbP21) corresponding to SPQ93225.1 was reported as bearing a chromosome segregation protein domain, which could be involved in pathogen proliferation or lifecycle transitions (Pérez-López et al., 2020).

Genes that are expressed consistently among replicates at more than one time-point may be relevant to initiating and maintaining infection. From the 22 transcripts found to be shared in the *P. brassicae* interaction with ‘Brutor’ at 14 and 21 dai, five corresponded to the previously discussed genes that were shared at both time-points and genotypes, and eight of the remaining transcripts were differentially expressed at 21 dai. Among the remaining transcripts, there were two protein kinases, and a NUDIX domain protein. From the two genes characterized as protein kinases, SPQ95766.1 corresponded to a *P. brassicae* effector named SSPbP22 that has been previously validated for kinase activity (Pérez-López et al., 2020). On the other hand, nudix effectors are common in bacteria, fungi and oomycetes, highlighting their importance in pathogenesis (Dong and Wang, 2016). Ectopic expression of the NUDIX domain effector Avr3b from the oomycete *Phytophthora sojae* reduces ROS, and its similarity with the host NUDIX regulator potentially results in negative regulation of the host immune response (Dong et al., 2011; Dong and Wang, 2016). A NUDIX hydrolase gene was also overexpressed in a pathobiome study where clubroot symptoms were severe (Daval et al., 2020).

### Orthologous Pathogen–Host Interactions

We compiled all non-redundant genes corresponding to secreted proteins from our differential and covariance analysis and submitted them to a database of pathogen–host interactions^5^, which provides information on genes proven to affect the interaction with the host (Urban et al., 2020). Among the 84 non-redundant secreted proteins determined from our pipeline (Supplementary Table 7), we selected those with a matching pathogen protein displaying a minimum e-value of 1e-5, and with a demonstrated effect on virulence upon its mutation (Table 2).

**TABLE 2:** Pathogen–host interactions (PHI)-base matches of non-redundant *P. brassicae* secreted protein transcripts, organized by phenotype after gene mutation/alteration.

Query ID	Query pfam domain	BLAST pathogen gene name hit	BLAST pathogen gene annotation	*e*-Value	PHI-base entry	Pathogen	Phenotype	Experimental technique	Host	Disease
SPQ94386.1	Peptidase M1 N-terminal domain/peptidase family M1 domain/leukotriene A4 hydrolase, C-terminal	PepN	Probable aminopeptidase	1.24E-19	PHI:3630	*Mycobacterium tuberculosis*	Increased virulence (hypervirulence)	Gene mutation	*Mus musculus*	Tuberculosis
SPQ97184.1	Protein kinase	CPK1	PKA catalytic subunit	4.56E-62	PHI:341	*Colletotrichum lag*enaria	Loss of pathogenicity	Gene disruption	*Cucumis sativus*	Anthracnose
SPQ97185.1	Protein kinase	CPK1	PKA catalytic subunit	1.77E-51	PHI:341	*Colletotrichum lagenaria*	Loss of pathogenicity	Gene disruption	*Cucumis sativus*	Anthracnose
SPQ95627.1	DnaJ central domain/DnaJ C terminal domain/DnaJ domain	mas5	Heat shock protein classified to Hsp40 group	1.21E-49	PHI:5394	*Beauveria bassiana*	Loss of pathogenicity	Gene mutation; gene complementation	*Galleria mellonella*	White muscardine disease
SPQ97950.1	WD domain, G-beta repeat	MoTup1	General transcriptional repressor	1.04E-08	PHI:4475	*Magnaporthe oryzae*	Loss of pathogenicity	Gene disruption; gene complementation	*Oryza sativa*, *Hordeum vulgare*	Rice blast
SPQ97562.1	WD domain, G-beta repeat	MoTup1	General transcriptional repressor	6.06E-05	PHI:4475	*Magnaporthe oryzae*	Loss of pathogenicity	Gene disruption; gene complementation	*Oryza sativa*, *Hordeum vulgare*	Rice blast
SPQ95942.1	Thioredoxin-like/glucosyltransferase 24/UDP-glucose:glycoprotein glucosyltransferase	KRE5	Beta-1,6-glucan synthesis	8.10E-172	PHI:6227	*Colletotrichum graminicola*	Reduced virulence	Altered gene expression/gene regulation: silencing	*Zea mays*	Stalk rot and leaf blight of maize
SPQ99747.1	Methyltransferase/SH3	AMT1	Arginine methyltransferase	2.17E-68	PHI:2351	*Fusarium graminearum*	Reduced virulence	Gene deletion: full; gene complementation	*Triticum aestivum*, *Zea mays*	Fusarium head blight
SPQ96145.1	Aldehyde dehydrogenase family	AldB_(PSPTO_2673)	Indole-3-acetaldehyde dehydrogenase	3.96E-55	PHI:7788	*Pseudomonas syringae*	Reduced virulence	Gene disruption	*Arabidopsis thaliana*	Bacterial speck of tomato
SPR01261.1	Serine carboxypeptidase	Rs-scp-1	Serine carboxypeptidases, esophageal gland-secreted protein	2.66E-41	PHI:7257	*Radopholus similis*	Reduced virulence	Altered gene expression/gene regulation: silencing	*Anthurium andraeanum*	Toppling or blackhead disease
SPR00473.1	Hsp70 protein	LHS1	Endoplasmic Reticulum Chaperone	1.91E-37	PHI:2058	*Magnaporthe oryzae*	Reduced virulence	Gene deletion: full; gene complementation	*Oryza sativa*	Rice blast
SPR01115.1	Protein kinase	PKA2	Cyclic AMP dependent protein kinase	1.98E-23	PHI:370	*Cryptococcus neoformans*	Reduced virulence	Gene deletion; gene complementation	*Mus musculus*	Cryptococcosis
SPQ94083.1	AMP-binding enzyme	fcs	Feruloyl-CoA synthetase involved in HCA degradation	2.31E-19	PHI:3419	*Ralstonia solanacearum*	Reduced virulence	Gene mutation: characterized; gene complementation	*Solanum lycopersicum*	Bacterial wilt
SPR00192.1	Leucine rich repeat	inlA	Internalin	1.67E-13	PHI:9300	*Listeria monocytogenes*	Reduced virulence	Gene mutation	*Homo sapiens*	Listeriosis
SPQ96032.1	Short chain dehydrogenase	fgm9_(FGSG_10989)	Hypothetical protein	2.86E-13	PHI:9041	*Fusarium graminearum*	Reduced virulence	Gene deletion: full	*Triticum aestivum*, *Zea mays*	Fusarium head blight, Ear rot in corn and yellow mold in peanuts
SPQ94523.1	Protein kinase	CZK3	MAP kinase kinase kinase	5.08E-13	PHI:296	*Cercospora zeae-maydis*	Reduced virulence	Gene disruption; gene complementation	*Zea mays*	Maize gray leaf spot
SPQ94819.1	Thioredoxin	Thioredoxin_1	Redox activity	3.26E-12	PHI:2644	*Salmonella enterica*	Reduced virulence	Gene deletion: full; gene complementation; chemical complementation	*Caenorhabditis elegans*	Food poisoning
SPR01650.1	NUDIX	PA4916	Unknown	4.50E-08	PHI:4718	*Pseudomonas aeruginosa*	Reduced virulence	Gene mutation	*Drosophila melanogaster*, *Mus musculus*	Nosocomial infection
SPQ98758.1	Protein tyrosine and serine/threonine kinase	cpkk2	Involved in the pheromone response pathway	7.24E-06	PHI:3988	*Cryphonectria parasitica*	Reduced virulence	Gene deletion; gene complementation	*Castanea dentata*	Chestnut blight
SPQ96649.1	Arsenite-resistance protein 2/Ras family/SERRATE/Ars2, N-terminal domain	GzC2H008	Transcription factor	1.92E-05	PHI:1348	*Fusarium graminearum*	Reduced virulence	Gene deletion	*Triticum aestivum*	Fusarium ear blight
SPR01410.1	Autophagy protein Atg8 ubiquitin like	atg8	Autophagy	4.01E-58	PHI:2497	*Ustilago maydis*	Reduced virulence loss of pathogenicity	Gene deletion: full	*Zea mays*	Maize smut disease

Twenty-one *P. brassicae* sequences corresponding to secreted proteins showed similarity to proteins from other pathogens where disruption or alteration of gene expression resulted in loss of pathogenicity, or increased or reduced virulence (Table 2). Some of these genes, which were discussed in the preceding sections, constitute important candidates for disruption in *P. brassicae*. Strategies including RNA interference (siRNA and dsRNA) have been used in plants infected by biotrophic and hemibiotrophic fungi to disrupt transcripts of important effectors (Nowara et al., 2010; Bharti et al., 2017; Zhu et al., 2017; Song and Thomma, 2018; Guo et al., 2019). The interfering RNA molecules transformed into the host are expected to be exchanged between host and pathogen through a vesicular system, resulting in degradation of the pathogen RNA target gene (Ghag, 2017; Rose et al., 2019). For example, disruption of CPK1 (protein kinase catalytic subunit) resulted in a loss of pathogenicity by *Colletotrichum lagenaria* on cucumber (Table 2). This protein was also disrupted through interfering RNAs using host-induced silencing in *P. striiformis* f. sp. *tritici*, through transformation of wheat plants, resulting in increased host resistance (Qi et al., 2018). Therefore, we infer that the *P. brassicae* query genes (SPQ97184.1, SPQ97185.1) could be suitable candidates for decreasing clubroot disease symptoms if RNA interference technology was applied.

Transcripts SPQ97950.1 and SPQ97562.1 matched *MoTup1* (*Magnaporthe oryzae* general transcriptional repressor *Tup1*), which upon mutagenesis presented loss of pathogenicity (Table 2). The most significant hit from the analysis was for SPQ95942.1, a gene with domains for thioredoxin and glucosyltransferase. Its matching hit in PHI-base is a Beta-1,6 glucan synthesis gene from the hemibiotroph *Colletotrichum graminicola*, which showed reduced virulence on *Zea mays* when RNAi was used (Oliveira-Garcia and Deising, 2016). Another candidate of interest is SPQ99747.1, which was similar to an arginine methyltransferase from *Fusarium graminearum.* The arginine methyltransferase (AMT1) from *F. graminearum* is a key gene in the methylation of ribonucleoprotein complexes that are exported from nucleus to cytoplasm for mRNA maturation, and therefore, its disruption results in decreased pathogen development and infection (Wang et al., 2012).

**FIGURE 5:**
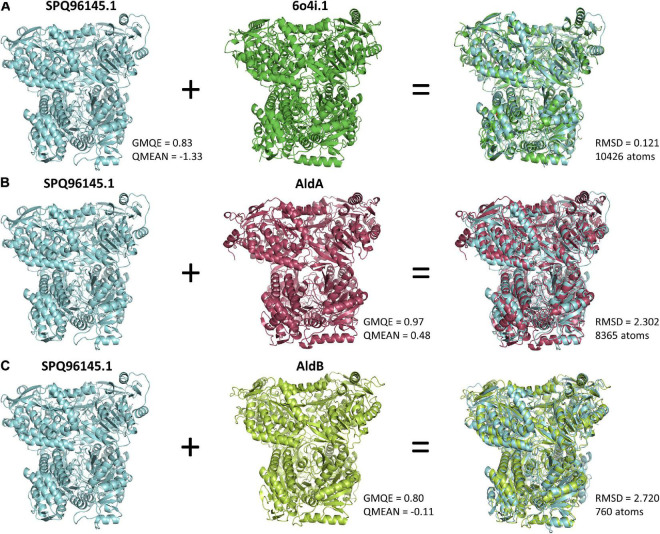
Predicted 3D structure of aldehyde dehydrogenases. **(A)** The aldehyde dehydrogenase from *P. brassicae* (SPQ96145.1) was predicted using Alpha-aminoadipic semialdehyde dehydrogenase (STML ID:6o4i.1.A – second structure from left to right) using Swiss-model. The structural alignment of the two structures can be seen on the third column. **(B,C)** Models for aldehyde dehydrogenases A and B (AldA and AldB -second column) from *P. syringae* which are involved in IAA synthesis, were aligned with the *P. brassicae* model, to produce the structural alignments seen on column 3. RMSD, root mean square deviation of all aligned atoms between the two structures, where a value of 0 is a perfect alignment. The number of aligned atoms is also reported for the three structural alignments. A Global Model Quality Estimation (GMQE) score closer to 1 represents more accuracy in the models, and a QMEAN value above –4 is indicative of good quality of the structure (https://swissmodel.expasy.org/docs/help).

Finally, one of the most interesting effector candidates may be SPQ96145.1, which showed similarity to an indole-3-acetaldehyde dehydrogenase from *Pseudomonas syringae.* We built a model of the protein (Figure 5), using an alpha-aminoadipic semialdehyde dehydrogenase (SMTL:6o4i.1) that resulted in a GMQE of 0.83 and a QMEAN of –1.33, showing high reliability for the model generated. Alignment of the generated model to the template (PDB accession 6o4i.1) resulted in a root-square mean deviation (RMSD) of 0.121 for a total of 10426 atoms aligned (Figure 5A). We also built models for aldehyde dehydrogenases A and B (Figures 5B,C). Aldehyde dehydrogenase B from *P. syringae*, was the match found on Table 2 for SPQ96145.1, while Aldehyde dehydrogenase A is another closely related aldehyde dehydrogenase from *P. syringae* involved in indole acetic acid (IAA) synthesis (McClerklin et al., 2017). Structural alignment of the SPQ9645.1 model to the AldA model resulted in a RMSD of 2.302 for a total of 8465 atoms, while a good RMSD for the alignment to AldB could only be achieved by aligning the closest atoms (760) between the structures using the cealigm option from PyMol. These results indicate good structure conservation between the *P. brassicae* protein and the *P. syringae* proteins, especially with AldA. *P. syringae* uses AldA and AldB to synthesize the auxin IAA, and it has been shown that disruption of these genes decreases virulence on *Arabidopsis thaliana* (McClerklin et al., 2017), with a higher disruption of IAA synthesis in *aldA* mutants. Moreover, the production of IAA by the pathogen increases virulence by disrupting SA-mediated defenses, which is complementary to increased susceptibility when auxin is generated by the host (McClerklin et al., 2017). Since auxin is one of the main players in clubroot development, but has generally been studied as a plant response hormone, a potential homologous mechanism to *P. syringae* (where not only the host but also *P. brassicae* aids in IAA production to promote virulence) could be in place. While the level of expression for this gene seems low in our study (Supplementary Table 6), a more complete study of the metabolic pathways leading to IAA production in *P. brassicae*, along with expression and functional analysis, would demonstrate the relevance of these key genes in infection, and open avenues for disruption of the disease.

### Validation and Evaluation

#### RNAseq Data Validation With Nanostrings

We used 28 predicted effector genes showing either significant differential expression at 21 dai between *P. brassicae* infecting the two *B. napus* genotypes, or showing low levels of covariance among biological replicates and consistent high expression at 14 and 21 dai for *P. brassicae* from either of the two genotypes (most of the genes selected are discussed throughout the manuscript).

Nanostrings nCounter technology is based on single RNA molecule detection without the need for amplification, using unique fluorescent barcodes bound to capture probes that bind to specific gene target templates (Nanostrings Technologies, 2021a). Once the probe complex is bound to the target, they can be captured on a streptavidin surface using a biotin tag from the probe, where they are immobilized for imaging. A scanner counts the number of each class of barcode, reflecting the number of RNA molecules from each gene.

After filtering a single gene with low Nanostring counts (<100 – SPQ94523), and one biological replicate from each genotype at 14 dai, flagged during mRNA positive control normalization, we compared average log_2_ RNAseq values with the average log_2_ Nanostring values. To establish the stability of five housekeeping genes, we plotted log_2_ counts of the five genes per sample to one random sample from either 14 or 21 dai, which confirmed that all genes were stable and could be used for differential expression analyses (Supplementary Figure 1).

**FIGURE 6:**
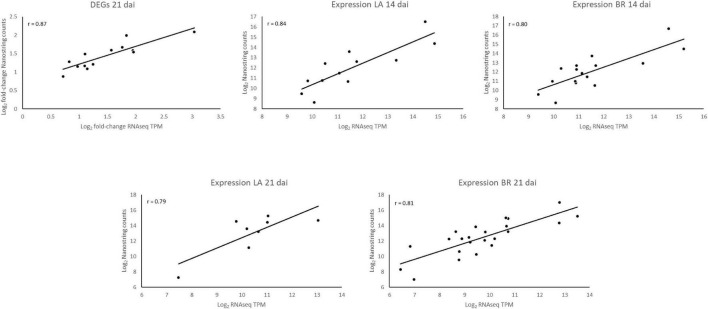
Validation of RNAseq data using nCounter Nanostrings technology. We evaluated 28 target genes which were either differentially expressed 21 dai or consistently expressed in both cultivars at 14 and 21 dai. As seen from the graphs, not all scatter plots have the same number of points, which reflects different amounts of genes evaluated under the different analyses according to the obtained results. (LA) *P. brassicae* genes coming from the interaction with ‘Laurentian,’ (BR) *P. brassicae* genes coming from the interaction with ‘Brutor,’ (DEGs) Differentially Expressed Genes.

We found a Pearson correlation of 0.87 at 21 dai when comparing the log_2_ fold changes between the *P. brassicae* RNAseq TPMs of the two genotypes with the log_2_ fold changes determined from the ratio counts with Nanostrings (Figure 6). The comparisons of the RNAseq results with the Nanostrings results for consistently expressed effectors at both 14 and 21 dai for both genotypes also showed correlations close to or >0.80 (Figure 6). This demonstrates the robustness of the RNAseq results, and shows that using Nanostrings as an alternative to qRT-PCR is a valid and less cumbersome approach to validate transcriptome results.

## Conclusion and Future Directions

Diverse prediction tools can sometimes provide different outcomes based on distinct algorithms. For example, most tools used in this study were predictive (e.g., HMMs or Neural Networks), but some other tools were annotated databases (e.g., ProSecKB). This will result in some gene annotation predictions not being confirmed by most tools, but allows the identification of more likely secreted effectors. While tools like SecretomeP were trained for mammalian sequences and have low reliability to predict leaderless secretory proteins in plants (Lonsdale et al., 2016), such tools have been used for the prediction of non-classical secretion in plant pathogenic fungi (Verma et al., 2016). SecretomeP is an important resource, since it predicted proteins as secreted in agreement with the protist secretome database (ProSecKB), with many of them not carrying a predicted signal peptide. This highlights the importance of looking at alternate routes of secretion and testing an array of tools for prediction.

While we only found a few transcripts differentially expressed from *P. brassicae* infecting either the resistant host ‘Laurentian’ or the susceptible host ‘Brutor,’ we observed a larger number of genes being consistently regulated among the three biological replicates coming from *P. brassicae* when infecting the susceptible cultivar. Likewise, we observed certain common genes coming from *P. brassicae* when infecting both genotypes (e.g., Supplementary Table 5). We believe that *P. brassicae* might use some of these core effectors as a general deployment strategy to promote infection, regardless of the host interaction.

Many of the predicted effectors (e.g., BSMT) have been shown as being key players in other studies (Ludwig-Müller et al., 2015; Pérez-López et al., 2020), and as such, represent good candidates for further study. We have selected the most relevant candidates from our study to summarize their importance in previous and future studies (Table 3). The 17 predicted secreted proteins are present in three other genome isolates: isolate Pb3 from Canada (Rolfe et al., 2016), ZJ-1 from China (Bi et al., 2019) and eH from Europe (Daval et al., 2019). These three isolates represent broad evolutionary divergence and have draft genomes which can be used for comparison. We found that all candidates selected are present in the other three genomes and have high levels of similarity (Table 3). A few hits have short unaligned regions (SPQ99629.1, SPQ96353.1, and SPQ97184.1) that may represent regions of variability, resulting in no alignment by the BLAST algorithm, or regions which might be absent in the target genome.

**TABLE 3:** Summary of most relevant *P. brassicae* effector candidates.

Gene ID	Annotation	Expression^1^	Functional validation^2^	Phenotype of homologous gene after mutation^3^	Putative role in breaking resistance	Gene present in other *P. brassicae* isolates^4^
SPQ95221.1	Hypothetical protein	Upregulated in BR at 14 dai and in LA at 21 dai	N/A	N/A	Unknown	Pb3 (99%), Pb_eH (100%), Pb_ZJ-1 (100%)
SPR00984.1	Hypothetical protein	Upregulated in LA at 21 dai	N/A	N/A	Unknown	Pb3 (99%), Pb_eH (100%), Pb_ZJ-1 (100%)
SPQ99629.1	30s ribosomal subunit 19	Upregulated in LA at 21 dai	N/A	N/A	Unknown	Pb3 (88% – 142 nucleotides unaligned), Pb_eH (100%), Pb_ZJ-1 (100%)
SPQ98385.1	E3 ubiquitin-protein ligase RING subunit	Upregulated in LA at 21 dai	N/A	N/A	Target host receptors for degradation (Yu et al., 2019)	Pb3 (100%), Pb_eH (100%), Pb_ZJ-1 (100%)
SPQ96771.1	S-phase kinase-associated protein 1	Upregulated in LA at 21 dai	N/A	N/A	Ubiquitination and degradation (Mena et al., 2020)	Pb3 (97%), Pb_eH (99%), Pb_ZJ-1 (100%)
SPQ93076.1	Benzoic acid/salicylic acid methyltransferase	High expression at 14 and 21 dai in both genotypes	Yes	N/A	Methylation of SA impairing plant response during clubroot disease (Ludwig-Müller et al., 2015), overexpression causes increases susceptibility to clubroot (Bulman et al., 2019; Djavaheri et al., 2019)	Pb3 (100%), Pb_eH (100%), Pb_ZJ-1 (100%)
SPR00473.1	Heat shock protein 70	High expression at 14 and 21 dai in both genotypes	N/A	Reduced virulence	Aids in trafficking and processing of secreted pathogen proteins from *P. falciparum* and *Magnaporthe oryzae* (Yi et al., 2009; Shonhai, 2010)	Pb3 (100%), Pb_eH (100%), Pb_ZJ-1 (100%)
SPQ96353.1	Peptide methionine sulfoxide reductase	High expression at 14 and 21 dai in both genotypes	N/A	N/A	Catalyzes the reduction of oxidized methionine back to methionine (Weissbach et al., 2002), and therefore potentially aids in protection of pathogen proteins from ROS (El Hassouni et al., 1999)	Pb3 (100% – 19 nucleotides unaligned), Pb_eH (100% – 19 nucleotides unaligned), Pb_ZJ-1 (100% – 19 nucleotides unaligned)
SPQ99289.1	Hypothetical protein	Upregulated in LA at 21 dai	N/A	N/A	Unknown	Pb3 (100%), Pb_eH (100%), Pb_ZJ-1 (100%)
SPR01261.1	Serine carboxypeptidase	Higher expression in BR at 14 dai and in LA at 21 dai	N/A	Reduced virulence	Resting spore germination (Feng et al., 2010)	Pb3 (99%), Pb_eH (100%), Pb_ZJ-1 (100%)
SPR01202.1	Stefin B	Higher expression in BR at 14 and 21 dai	N/A	N/A	Protease inhibitor against host proteases (Gumtow et al., 2018)	Pb3 (100%), Pb_eH (100%), Pb_ZJ-1 (100%)
SPQ95766.1	Protein kinase	Higher expression in BR at 14 dai and in LA at 21 dai	Yes	N/A	Potentially affecting cell cycle progression (Pérez-López et al., 2020)	Pb3 (100%), Pb_eH (100%), Pb_ZJ-1 (100%)
SPQ97184.1	Protein kinase	Upregulated in LA at 21 dai	N/A	Loss of pathogenicity	Germination, appressorium formation and infectious growth in *Colletotrichum lagenarium* (Yamauchi et al., 2004)	Pb3 (99% – 46 nucleotides unaligned), Pb_eH (100%), Pb_ZJ-1 (100%)
SPQ97185.1	Protein kinase	Upregulated in LA at 21 dai	N/A	Loss of pathogenicity	Germination, appressorium formation and infectious growth in *C. lagenarium* (Yamauchi et al., 2004)	Pb3 (99%), Pb_eH (100%), Pb_ZJ-1 (100%)
SPQ95942.1	Thioredoxin-like/glucosyltransferase 24	Low expression (TPM < 100) at 14 and 21 dai in both genotypes	N/A	Reduced virulence	Cell wall/appressorium rigidity (Oliveira-Garcia and Deising, 2016)	Pb3 (100%), Pb_eH (100%), Pb_ZJ-1 (98%)
SPQ99747.1	Methyltransferase	Higher expression in LA at 21 dai	N/A	Reduced virulence	The arginine methyltransferase from *F. graminearum* methylates ribonucleoprotein complexes exported from the nucleus to the cytoplasm for mRNA maturation (Wang et al., 2012)	Pb3 (99%), Pb_eH (100%), Pb_ZJ-1 (99%)
SPQ96145.1	Indole-3-acetaldehyde dehydrogenase	Low expression (TPM < 100) at 21 dai in both genotypes, and no expression detected at 14 dai	N/A	Reduced virulence	Synthesis of the auxin IAA by *P. syringae* (McClerklin et al., 2017). Increases of auxin during *P. brassicae* infection are central to disease development.	Pb3 (100%), Pb_eH (100%), Pb_ZJ-1 (100%)

*^1^Upregulation refers to a significant difference in expression. Expression values can be seen in Table 1 and Supplementary Table 6.*

*^2^Functional validation in the context of the clubroot pathosystem.*

*^3^Refers to the comparison made with PHI-base in Table 2.*

*^4^Genome isolates correspond to pathotypes Pb3 (Rolfe et al., 2016), ZJ-1 (Bi et al., 2019), and eH (Daval et al., 2019). Unaligned nucleotides correspond to regions from the query without a High-Scoring Pair (HSP), during BLAST analysis.*

The presence of these putative secreted proteins in a wide variety of genomes highlights the importance of validating their roles using techniques like RNA interference. The candidates can also be used for designing high-throughput yeast-two hybrid assays to discover potential host targets of virulence. Future disruption of effector-receptor associations holds promise for understanding the basis of clubroot disease.

## Data Availability Statement

Publicly available datasets were analyzed in this study. This data can be found here: https://www.ncbi.nlm.nih.gov/sra/?term=PRJNA597078. Sequencing reads used in this study correspond to a previous study Galindo-González et al. (2020). The reads are deposited in the NCBI Sequence Read Archive (SRA) under accession number PRJNA597078.

## Author Contributions

LG-G designed and conducted all the experiments, performed all analyses, and wrote the manuscript. S-FH helped secure project funding and contributed to the original experimental context. SS provided project guidance and edited several versions of the manuscript. All authors contributed to the article and approved the submitted version.

## Conflict of Interest

The authors declare that the research was conducted in the absence of any commercial or financial relationships that could be construed as a potential conflict of interest.

## Publisher’s Note

All claims expressed in this article are solely those of the authors and do not necessarily represent those of their affiliated organizations, or those of the publisher, the editors and the reviewers. Any product that may be evaluated in this article, or claim that may be made by its manufacturer, is not guaranteed or endorsed by the publisher.

## Footnotes

1
https://www.bioinformatics.babraham.ac.uk/projects/fastqc/

2
http://www.bioinformatics.nl/cgi-bin/emboss/fuzzpro

3
https://swissmodel.expasy.org/docs/help

4
https://www.ncbi.nlm.nih.gov/genome/browse#!/eukaryotes/38756/

5
http://www.phi-base.org/

## References

[B1] Alamuri P. Maier R. J. (2004). Methionine sulphoxide reductase is an important antioxidant enzyme in the gastric pathogen *Helicobacter pylori*. *Mol. Microbiol.* 53 1397–1406. 10.1111/j.1365-2958.2004.04190.x 15387818

[B2] Almagro Armenteros J. J. Sønderby C. K. Sønderby S. K. Nielsen H. Winther O. (2017). DeepLoc: prediction of protein subcellular localization using deep learning. *Bioinformatics* 33 3387–3395. 10.1093/bioinformatics/btx431 29036616

[B3] Altschul S. Gish W. Miller W. Myers E. Lipman D. (1990). Basic local alignment search tool. *J. Mol. Biol.* 215 403–410. 2231712 10.1016/S0022-2836(05)80360-2

[B4] Armenteros J. J. A. Salvatore M. Emanuelsson O. Winther O. Von Heijne G. Elofsson A. (2019). Detecting sequence signals in targeting peptides using deep learning. *Life Sci. Alliance* 2:e201900429. 10.26508/lsa.201900429 31570514 PMC6769257

[B5] Badstöber J. Gachon C. M. M. Ludwig-Müller J. Sandbichler A. M. Neuhauser S. (2020). Demystifying biotrophs: FISHing for mRNAs to decipher plant and algal pathogen–host interaction at the single cell level. *Sci. Rep.* 10:14269. 10.1038/S41598-020-70884-4 32868853 PMC7459097

[B6] Bendtsen J. D. Jensen L. J. Blom N. von Heijne G. Brunak S. (2004). Feature-based prediction of non-classical and leaderless protein secretion. *Protein Eng. Des. Sel.* 17 349–356. 10.1093/protein/gzh037 15115854

[B7] Bharti P. Jyoti P. Kapoor P. Sharma V. Shanmugam V. Yadav S. K. (2017). Host-induced silencing of pathogenicity genes enhances resistance to *Fusarium oxysporum* wilt in tomato. *Mol. Biotechnol.* 59 343–352. 10.1007/s12033-017-0022-y 28674943

[B8] Bi K. Chen T. He Z. Gao Z. Zhao Y. Liu H. (2019). Comparative genomics reveals the unique evolutionary status of *Plasmodiophora brassicae* and the essential role of GPCR signaling pathways. *Phytopathol. Res.* 1:12. 10.1186/S42483-019-0018-6

[B9] Bode W. Engh R. Musil D. Thiele U. Huber R. Karshikov A. (1988). The 2.0 A X-ray crystal structure of chicken egg white cystatin and its possible mode of interaction with cysteine proteinases. *EMBO J.* 7 2593–2599. 10.1002/j.1460-2075.1988.tb03109.x 3191914 PMC457133

[B10] Bulman S. Richter F. Marschollek S. Benade F. Jülke S. Ludwig-Müller J. (2019). *Arabidopsis thaliana* expressing PbBSMT, a gene encoding a SABATH-type methyltransferase from the plant pathogenic protist *Plasmodiophora brassicae*, show leaf chlorosis and altered host susceptibility. *Plant Biol.* 21 120–130. 10.1111/plb.12728 29607585

[B11] Bulman S. Siemens J. Ridgway H. J. Eady C. Conner A. J. (2006). Identification of genes from the obligate intracellular plant pathogen, *Plasmodiophora brassicae*. *FEMS Microbiol. Lett.* 264 198–204. 10.1111/j.1574-6968.2006.00466.x 17064373

[B12] Cao T. Manolii V. P. Zhou Q. Hwang S. F. Strelkov S. E. (2020). Effect of canola (*Brassica napus*) cultivar rotation on *Plasmodiophora brassicae* pathotype composition. *Can. J. Plant Sci.* 100 218–225. 10.1139/cjps-2019-0126 33356898

[B13] Chen J. Pang W. Chen B. Zhang C. Piao Z. (2016). Transcriptome analysis of *Brassica rapa* near-isogenic lines carrying clubroot-resistant and susceptible alleles in response to *Plasmodiophora brassicae* during early infection. *Front. Plant Sci.* 6:1183. 10.3389/fpls.2015.01183 26779217 PMC4700149

[B14] Ciaghi S. Schwelm A. Neuhauser S. (2019). Transcriptomic response in symptomless roots of clubroot infected kohlrabi (*Brassica oleracea* var. gongylodes) mirrors resistant plants. *BMC Plant Biol.* 19:288. 10.1186/s12870-019-1902-z 31262271 PMC6604361

[B15] Conesa A. Götz S. (2008). Blast2GO: a comprehensive suite for functional analysis in plant genomics. *Int. J. Plant Genomics* 2008:619832. 10.1155/2008/619832 18483572 PMC2375974

[B16] Conesa A. Gotz S. Garcia-Gomez J. M. Terol J. Talon M. Robles M. (2005). Blast2GO: a universal tool for annotation, visualization and analysis in functional genomics research. *Bioinformatics* 21 3674–3676. 10.1093/bioinformatics/bti610 16081474

[B17] Dalman K. Himmelstrand K. Olson Å Lind M. Brandström-Durling M. Stenlid J. (2013). A genome-wide association study identifies genomic regions for virulence in the non-model organism *Heterobasidion annosum* s.s. *PLoS One* 8:e53525. 10.1371/journal.pone.0053525 23341945 PMC3547014

[B18] Daval S. Belcour A. Gazengel K. Legrand L. Gouzy J. Cottret L. (2019). Computational analysis of the *Plasmodiophora brassicae* genome: mitochondrial sequence description and metabolic pathway database design. *Genomics* 111 1629–1640. 10.1016/J.YGENO.2018.11.013 30447277

[B19] Daval S. Gazengel K. Belcour A. Linglin J. Guillerm-Erckelboudt A. Y. Sarniguet A. (2020). Soil microbiota influences clubroot disease by modulating *Plasmodiophora brassicae* and *Brassica napus* transcriptomes. *Microb. Biotechnol.* 13 1648–1672. 10.1111/1751-7915.13634 32686326 PMC7415369

[B20] Denkel L. A. Horst S. A. Rouf S. F. Kitowski V. Böhm O. M. Rhen M. (2011). Methionine sulfoxide reductases are wssential for virulence of *Salmonella typhimurium*. *PLoS One* 6:e26974. 10.1371/journal.pone.0026974 22073230 PMC3206869

[B21] Dixon G. R. (2009). The occurrence and economic impact of *Plasmodiophora brassicae* and clubroot disease. *J. Plant Growth Regul.* 28 194–202. 10.1007/s00344-009-9090-y

[B22] Djavaheri M. Ma L. Klessig D. F. Mithöfer A. Mithöfer M. Gropp G. (2019). Mimicking the host regulation of salicylic acid: a virulence strategy by the clubroot pathogen *Plasmodiophora brassicae*. *Mol. Plant Microbe Interact.* 32 296–305. 10.1094/MPMI-07-18-0192-R 30199341

[B23] Dodds P. N. Rathjen J. P. (2010). Plant immunity: towards an integrated view of plant-pathogen interactions. *Nat. Rev. Genet.* 11 539–548. 10.1038/nrg2812 20585331

[B24] Donald C. Porter I. (2009). Integrated control of clubroot. *J. Plant Growth Regul.* 28 289–303. 10.1007/s00344-009-9094-7

[B25] Dong S. Wang Y. (2016). Nudix effectors: a common weapon in the arsenal of plant pathogens. *PLoS Pathog.* 12:e1005704. 10.1371/journal.ppat.1005704 27737001 PMC5063578

[B26] Dong S. Yin W. Kong G. Yang X. Qutob D. Chen Q. (2011). *Phytophthora sojae* avirulence effector Avr3b is a secreted NADH and ADP-ribose pyrophosphorylase that modulates plant immunity. *PLoS Pathog.* 7:e1002353. 10.1371/journal.ppat.1002353 22102810 PMC3213090

[B27] El Hassouni M. Chambost J. P. Expert D. Van Gijsegem F. Barras F. (1999). The minimal gene set member *msrA*, encoding peptide methionine sulfoxide reductase, is a virulence determinant of the plant pathogen *Erwinia chrysanthemi*. *Proc. Natl. Acad. Sci. U.S.A.* 96 887–892. 10.1073/pnas.96.3.887 9927663 PMC15320

[B28] Feng J. I. E. Hwang R. U. Hwang S. Strelkov S. E. Gossen B. D. Zhou Q. (2010). Molecular characterization of a serine protease Pro1 from *Plasmodiophora brassicae* that stimulates resting spore germination. *Mol. Plant Pathol.* 11 503–512. 10.1111/J.1364-3703.2010.00623.X 20618708 PMC6640502

[B29] Galindo-González L. Manolii V. Hwang S. F. Strelkov S. E. (2020). Response of *Brassica napus* to *Plasmodiophora brassicae* involves salicylic acid-mediated immunity: an RNA-Seq-based study. *Front. Plant Sci.* 11:1025. 10.3389/fpls.2020.01025 32754180 PMC7367028

[B30] Ghag S. B. (2017). Host induced gene silencing, an emerging science to engineer crop resistance against harmful plant pathogens. *Physiol. Mol. Plant Pathol.* 100 242–254. 10.1016/j.pmpp.2017.10.003

[B31] Gíslason M. H. Nielsen H. Almagro Armenteros J. J. Johansen A. R. (2021). Prediction of GPI-anchored proteins with pointer neural networks. *Curr. Res. Biotechnol.* 3 6–13. 10.1016/j.crbiot.2021.01.001

[B32] Gout L. Fudal I. Kuhn M.-L. Blaise F. Eckert M. Cattolico L. (2006). Lost in the middle of nowhere: the AvrLm1 avirulence gene of the Dothideomycete *Leptosphaeria maculans*. *Mol. Microbiol.* 60 67–80. 10.1111/j.1365-2958.2006.05076.x 16556221

[B33] Gumtow R. Wu D. Uchida J. Tian M. (2018). A *Phytophthora palmivora* extracellular cystatin-like protease inhibitor targets papain to contribute to virulence on papaya. *Mol. Plant Microbe Interact.* 31 363–373. 10.1094/MPMI-06-17-0131-FI 29068239

[B34] Guo X. Y. Li Y. Fan J. Xiong H. Xu F. X. Shi J. (2019). Host-induced gene silencing of *Moap1* confers broad-spectrum resistance to *Magnaporthe oryzae*. *Front. Plant Sci.* 10:433. 10.3389/fpls.2019.00433 31024598 PMC6465682

[B35] Hollman K. B. Hwang S. F. Manolii V. P. Strelkov S. E. (2021). Pathotypes of *Plasmodiophora brassicae* collected from clubroot resistant canola (*Brassica napus* L.) cultivars in western Canada in 2017-2018. *Can. J. Plant Pathol.* 43 622–630. 10.1080/07060661.2020.1851893

[B36] Horton P. Park K. J. Obayashi T. Fujita N. Harada H. Adams-Collier C. J. (2007). WoLF PSORT: protein localization predictor. *Nucleic Acids Res.* 35 W585–W587. 10.1093/nar/gkm259 17517783 PMC1933216

[B37] Hossain M. M. Pérez-López E. Todd C. D. Wei Y. Bonham-Smith P. C. (2021). Endomembrane-targeting *Plasmodiophora brassicae* effectors modulate PAMP triggered immune responses in plants. *Front. Microbiol.* 12:651279. 10.3389/fmicb.2021.651279 34276588 PMC8282356

[B38] Hunter S. Apweiler R. Attwood T. K. Bairoch A. Bateman A. Binns D. (2009). InterPro: the integrative protein signature database. *Nucleic Acids Res.* 37 D211–D215. 10.1093/nar/gkn785 18940856 PMC2686546

[B39] Hwang S. F. Howard R. J. Strelkov S. E. Gossen B. D. Peng G. (2014). Management of clubroot (*Plasmodiophora brassicae*) on canola (*Brassica napus*) in western Canada. *Can. J. Plant Pathol.* 36 49–65. 10.1080/07060661.2013.863806

[B40] Hwang S.-F. Strelkov S. E. Feng J. Gossen B. D. Howard R. J. (2012). Pathogen profile *Plasmodiophora brassicae*: a review of an emerging pathogen of the Canadian canola (*Brassica napus*) crop. *Mol. Plant Pathol.* 13 105–113. 10.1111/J.1364-3703.2011.00729.X 21726396 PMC6638701

[B41] Jahn L. Mucha S. Bergmann S. Horn C. Staswick P. Steffens B. (2013). The clubroot pathogen (*Plasmodiophora brassicae*) influences auxin signaling to regulate auxin homeostasis in *Arabidopsis*. *Plants* 1 726–749. 10.3390/plants2040726 27137401 PMC4844388

[B42] Joshi V. Upadhyay A. Kumar A. Mishra A. (2017). Gp78 E3 ubiquitin ligase: essential functions and contributions in proteostasis. *Front. Cell. Neurosci.* 11:259. 10.3389/fncel.2017.00259 28890687 PMC5575403

[B43] Kageyama K. Asano ÆT. (2009). Life cycle of *Plasmodiophora brassicae*. *J. Plant Growth Regul.* 28 203–211. 10.1007/s00344-009-9101-z

[B44] Kim K. T. Jeon J. Choi J. Cheong K. Song H. Choi G. (2016). Kingdom-wide analysis of fungal small secreted proteins (SSPs) reveals their potential role in host association. *Front. Plant Sci.* 7:186. 10.3389/fpls.2016.00186 26925088 PMC4759460

[B45] Kim S. T. Yu S. Kim S. G. Kim H. J. Kang S. Y. Hwang D. H. (2004). Proteome analysis of rice blast fungus (*Magnaporthe grisea*) proteome during appressorium formation. *Proteomics* 4 3579–3587. 10.1002/pmic.200400969 15378734

[B46] Kumar S. Bhardwaj T. R. Prasad D. N. Singh R. K. (2018). Drug targets for resistant malaria: historic to future perspectives. *Biomed. Pharmacother.* 104 8–27. 10.1016/j.biopha.2018.05.009 29758416

[B47] Lemarie S. Robert-seilaniantz A. Lariagon C. Lemoine J. Manzanares-Dauleux M. J. Gravot A. (2015). Both the jasmonic acid and the salicylic acid pathways contribute to resistance to the biotrophic clubroot agent *Plasmodiophora brassicae* in *Arabidopsis*. *Plant Cell Physiol.* 56 2158–2168. 10.1093/pcp/pcv127 26363358

[B48] Li H. Handsaker B. Wysoker A. Fennell T. Ruan J. Homer N. (2009). The sequence alignment/map format and SAMtools. *Bioinformatics* 25 2078–2079. 10.1093/bioinformatics/btp352 19505943 PMC2723002

[B49] Liu L. Qin L. Zhou Z. Hendriks W. G. H. M. Liu S. Wei Y. (2020). Refining the life cycle of *Plasmodiophora brassicae*. *Phytopathology* 110 1704–1712. 10.1094/PHYTO-02-20-0029-R 32407251

[B50] Lonsdale A. Davis M. J. Doblin M. S. Bacic A. (2016). Better than nothing? Limitations of the prediction tool secretomeP in the search for leaderless secretory proteins (LSPs) in plants. *Front. Plant Sci.* 7. 10.3389/fpls.2016.01451 27729919 PMC5037178

[B51] Love M. I. Huber W. Anders S. (2014). Moderated estimation of fold change and dispersion for RNA-seq data with DESeq2. *Genome Biol.* 15:550. 10.1186/s13059-014-0550-8 25516281 PMC4302049

[B52] Ludwig-Müller J. Auer S. Jülke S. Marschollek S. (2017). “Manipulation of auxin and cytokinin balance during the *Plasmodiophora brassicae*-*Arabidopsis thaliana* interaction,” in *Auxins and Cytokinins in Plant Biology*, eds Dandekar T. Neaseem M. (Totowa, NJ: Humana Press), 41–60. 10.1007/978-1-4939-6831-2 28265986

[B53] Ludwig-Müller J. Jülke S. Geiß K. Richter F. Mithöfer A. Šola I. (2015). A novel methyltransferase from the intracellular pathogen *Plasmodiophora brassicae* methylates salicylic acid. *Mol. Plant Pathol.* 16 349–364. 10.1111/mpp.12185 25135243 PMC6638400

[B54] Marti M. Good R. T. Rug M. Knuepfer E. Cowman A. F. (2004). Targeting malaria virulence and remodeling proteins to the host erythrocyte. *Science* 306 1930–1933. 10.1126/science.1102452 15591202

[B55] McClerklin S. A. Lee S. G. Nwumeh R. Jez J. M. Kunkel B. N. (2017). Indole-3-acetaldehyde dehydrogenase-dependent auxin synthesis contributes to virulence of *Pseudomonas syringae* strain DC3000. *bioRxiv* [Preprint] 1–24. 10.1101/173302 PMC5766252 29293681

[B56] Mena E. L. Jevtić P. Greber B. J. Gee C. L. Lew B. G. Akopian D. (2020). Structural basis for dimerization quality control. *Nature* 586 452–456. 10.1038/s41586-020-2636-7 32814905 PMC8024055

[B57] Mukhi N. Gorenkin D. Banfield M. J. (2020). Exploring folds, evolution and host interactions: understanding effector structure/function in disease and immunity. *New Phytol.* 227 326–333. 10.1111/nph.16563 32239533

[B58] Nagata K. Kudo N. Abe K. Arai S. Tanokura M. (2000). Three-dimensional solution structure of oryzacystatin-I, a cysteine proteinase inhibitor of the rice, *Oryza sativa* L. japonica. *Biochemistry* 39 14753–14760. 10.1021/bi0006971 11101290

[B59] Nanostrings Technologies (2011). *nCounter^®^ Expression CodeSet Design Manual.* Seattle, WA. Available online at: www.nanostring.com (accessed June 3, 2021).

[B60] Nanostrings Technologies (2021b). *nCounter Gene Expression CodeSet RNA Hybridization Protocol.* Seattle, WA: Nanostrings Technologies.

[B61] Nanostrings Technologies (2021a). *Nanostrings nCounter.* Available online at: https://www.bioxpedia.com/nanostring-ncounter-technology/#:~:text=NanoString is a robust and reproducible technology and, detection sensitivity down to 1 copy per cell (accessed June 15, 2021).

[B62] Nielsen H. (2017). “Predicting secretory proteins with signaIP,” in *Methods in Molecular Biology*, ed. Kihara D. (New York, NY: Humana Press Inc.), 59–73. 10.1007/978-1-4939-7015-5_6 28451972

[B63] Nogueira-Lopez G. Greenwood D. R. Middleditch M. Winefield C. Eaton C. Steyaert J. M. (2018). The apoplastic secretome of *Trichoderma virens* during interaction with maize roots shows an inhibition of plant defence and scavenging oxidative stress secreted proteins. *Front. Plant Sci.* 9:409. 10.3389/fpls.2018.00409 29675028 PMC5896443

[B64] Nowara D. Schweizer P. Gay A. Lacomme C. Shaw J. Ridout C. (2010). HIGS: host-induced gene silencing in the obligate biotrophic fungal pathogen *Blumeria graminis*. *Plant Cell* 22 3130–3141. 10.1105/tpc.110.077040 20884801 PMC2965548

[B65] Oliveira-Garcia E. Deising H. B. (2016). Attenuation of PAMP-triggered immunity in maize requires down-regulation of the key β-1,6-glucan synthesis genes KRE5 and KRE6 in biotrophic hyphae of *Colletotrichum graminicola*. *Plant J.* 87 355–375. 10.1111/tpj.13205 27144995

[B66] Pérez-López E. Hossain M. M. Tu J. Waldner M. Todd C. D. Kusalik A. J. (2020). Transcriptome analysis identifies *Plasmodiophora brassicae* secondary infection effector candidates. *J. Eukaryot. Microbiol.* 67 337–351. 10.1111/jeu.12784 31925980 PMC7317818

[B67] Pérez-López E. Hossain M. M. Wei Y. Todd C. D. Bonham-Smith P. C. (2021). A clubroot pathogen effector targets cruciferous cysteine proteases to suppress plant immunity. *Virulence* 12 2327–2340. 10.1080/21505594.2021.1968684 34515618 PMC8451464

[B68] Pérez-López E. Waldner M. Hossain M. Kusalik A. J. Wei Y. Bonham-smith P. C. (2018). Identification of *Plasmodiophora brassicae* effectors — a challenging goal. *Virulence* 9 1344–1353. 10.1080/21505594.2018.1504560 30146948 PMC6177251

[B69] Pierleoni A. Martelli P. Casadio R. (2008). PredGPI: a GPI-anchor predictor. *BMC Bioinformatics* 9:392. 10.1186/1471-2105-9-392 18811934 PMC2571997

[B70] Powell B. Amerishetty V. Meinken J. Knott G. Yu F. Cooper C. (2016). ProtSecKB: the protist secretome and subcellular proteome knowledgebase. *Comput. Mol. Biol.* 6 1–12. 10.5376/cmb.2016.06.0004

[B71] Prakash C. Manjrekar J. Chattoo B. B. (2016). Skp1, a component of E3 ubiquitin ligase, is necessary for growth, sporulation, development and pathogenicity in rice blast fungus (*Magnaporthe oryzae*). *Mol. Plant Pathol.* 17 903–919. 10.1111/mpp.12336 26575697 PMC6638394

[B72] Prerostova S. Dobrev P. I. Konradyova V. Knirsch V. Gaudinova A. Kramna B. (2018). Hormonal responses to *Plasmodiophora brassicae* infection in *Brassica napus* cultivars differing in their pathogen resistance. *Int. J. Mol. Sci.* 19:4024. 10.3390/ijms19124024 30551560 PMC6321006

[B73] Qi T. Zhu X. Tan C. Liu P. Guo J. Kang Z. (2018). Host-induced gene silencing of an important pathogenicity factor *PsCPK1* in *Puccinia striiformis* f. sp. *tritici* enhances resistance of wheat to stripe rust. *Plant Biotechnol. J.* 16 797–807. 10.1111/pbi.12829 28881438 PMC5814584

[B74] Quevillon E. Silventoinen V. Pillai S. Harte N. Mulder N. Apweiler R. (2005). InterProScan: protein domains identifier. *Nucleic Acids Res.* 33 W116–W120. 10.1093/nar/gki442 15980438 PMC1160203

[B75] Quinlan A. R. Hall I. M. (2010). BEDTools: a flexible suite of utilities for comparing genomic features. *Bioinformatics* 26 841–842. 10.1093/bioinformatics/btq033 20110278 PMC2832824

[B76] Roberts A. Pachter L. (2013). Streaming fragment assignment for real-time analysis of sequencing experiments. *Nat. Methods* 10 71–73. 10.1038/nmeth.2251 23160280 PMC3880119

[B77] Rolfe S. A. Strelkov S. E. Links M. G. Clarke W. E. Robinson S. J. Djavaheri M. (2016). The compact genome of the plant pathogen *Plasmodiophora brassicae* is adapted to intracellular interactions with host *Brassica* spp. *BMC Genomics* 17:272. 10.1186/s12864-016-2597-2 27036196 PMC4815078

[B78] Rose L. E. Overdijk E. J. R. van Damme M. (2019). Small RNA molecules and their role in plant disease. *Eur. J. Plant Pathol.* 154 115–128. 10.1007/s10658-018-01614-w 30880875 PMC6394340

[B79] Schuller A. Kehr J. Ludwig-Müller J. (2014). Laser microdissection coupled to transcriptional profiling of *Arabidopsis* roots inoculated by *Plasmodiophora brassicae* indicates a role for brassinosteroids in clubroot formation. *Plant Cell Physiol.* 55 392–411. 10.1093/pcp/pct174 24285749

[B80] Schwelm A. Fogelqvist J. Knaust A. Jülke S. Lilja T. (2015). The *Plasmodiophora brassicae* genome reveals insights in its life cycle and ancestry of chitin synthases. *Nature* 5:11153. 10.1038/srep11153 26084520 PMC4471660

[B81] Selin C. de Kievit T. R. Belmonte M. F. Fernando W. G. D. Monaghan J. (2016). Elucidating the role of effectors in plant-fungal interactions?: progress and challenges. *Front. Microbiol.* 7:600. 10.3389/fmicb.2016.00600 27199930 PMC4846801

[B82] Shonhai A. (2010). Plasmodial heat shock proteins: targets for chemotherapy. *FEMS Immunol. Med. Microbiol.* 58 61–74. 10.1111/j.1574-695X.2009.00639.x 20041948

[B83] Siemens J. Graf H. Bulman S. In O. (2009). Monitoring expression of selected *Plasmodiophora brassicae* genes during clubroot development in *Arabidopsis thaliana*. *Plant Pathol.* 58 130–136. 10.1111/j.1365-3059.2008.01943.x

[B84] Singh V. K. Singh K. Baum K. (2018). The role of methionine sulfoxide reductases in oxidative stress tolerance and virulence of *Staphylococcus aureus* and other bacteria. *Antioxidants* 7:128. 10.3390/antiox7100128 30274148 PMC6210949

[B85] Song Y. Thomma B. P. H. J. (2018). Host-induced gene silencing compromises *Verticillium wilt* in tomato and *Arabidopsis*. *Mol. Plant Pathol.* 19 77–89. 10.1111/mpp.12500 27749994 PMC6638114

[B86] Sonnhammer E. L. L. Von Heijne G. Krogh A. (1998). “A hidden markov model for predicting transmembrane helices in protein sequences,” in *Proceedings of the 6th International Conference on Intelligent Systems for Molecular Biology: ISMB’98.* Available online at: www.aaai.org (accessed April 16, 2021). 9783223

[B87] Sperschneider J. Dodds P. N. Gardiner D. M. Singh K. B. Taylor J. M. (2018a). Improved prediction of fungal effector proteins from secretomes with EffectorP 2.0. *Mol. Plant Pathol.* 19 2094–2110. 10.1111/mpp.12682 29569316 PMC6638006

[B88] Sperschneider J. Dodds P. N. Singh K. B. Taylor J. M. (2018b). ApoplastP: prediction of effectors and plant proteins in the apoplast using machine learning. *New Phytol.* 217 1764–1778. 10.1111/nph.14946 29243824

[B89] Sperschneider J. Gardiner D. M. Dodds P. N. Tini F. Covarelli L. Singh K. B. (2015). Effector P: predicting fungal effector proteins from secretomes using machine learning. *New Phytol.* 210 743–761. 10.1111/nph.13794 26680733

[B90] Stjelja S. Fogelqvist J. Tellgren-Roth C. Dixelius C. (2019). The architecture of the *Plasmodiophora brassicae* nuclear and mitochondrial genomes. *Sci. Rep.* 9:15753. 10.1038/s41598-019-52274-7 31673019 PMC6823432

[B91] Strelkov S. E. Hwang S. F. Manolii V. P. Cao T. Fredua-Agyeman R. Harding M. W. (2018). Virulence and pathotype classification of *Plasmodiophora brassicae* populations collected from clubroot resistant canola (*Brassica napus*) in Canada. *Can. J. Plant Pathol.* 40 284–298. 10.1080/07060661.2018.1459851

[B92] Strelkov S. E. Hwang S. Manolii V. P. Cao T. Feindel D. (2016). Emergence of new virulence phenotypes of *Plasmodiophora brassicae* on canola (*Brassica napus*) in Alberta, Canada. *Eur. J. Plant Pathol.* 145 517–529. 10.1007/s10658-016-0888-8

[B93] Toruño T. Y. Stergiopoulos I. Coaker G. (2016). Plant-pathogen effectors: cellular probes interfering with plant defenses in spatial and temporal manners. *Annu. Rev. Phytopathol.* 54 419–441. 10.1146/annurev-phyto-080615-100204 27359369 PMC5283857

[B94] Turk V. Bode W. (1991). The cystatins: protein inhibitors of cysteine proteinases. *FEBS Lett.* 285 213–219. 10.1016/0014-5793(91)80804-C 1855589

[B95] Urban M. Cuzick A. Seager J. Wood V. Rutherford K. Venkatesh S. Y. (2020). PHI-base: the pathogen-host interactions database. *Nucleic Acids Res.* 48 D613–D620. 10.1093/nar/gkz904 31733065 PMC7145647

[B96] Verma S. Gazara R. K. Nizam S. Parween S. Chattopadhyay D. Verma P. K. (2016). Draft genome sequencing and secretome analysis of fungal phytopathogen Ascochyta rabiei provides insight into the necrotrophic effector repertoire. *Sci. Rep.* 6 1–14. 10.1038/srep24638 27091329 PMC4835772

[B97] Wang B. Song N. Tang C. Ma J. Wang N. Sun Y. (2019). PsRPs26, a 40S ribosomal protein subunit, regulates the growth and pathogenicity of *Puccinia striiformis* f. sp. tritici. *Front. Microbiol.* 10:968. 10.3389/fmicb.2019.00968 31134016 PMC6523408

[B98] Wang G. Wang C. Hou R. Zhou X. Li G. Zhang S. (2012). The *AMT1* arginine methyltransferase gene is important for plant infection and normal hyphal growth in *Fusarium graminearum*. *PLoS One* 7:e38324. 10.1371/journal.pone.0038324 22693618 PMC3365026

[B99] Wang Y. Wang Y. Wang Y. (2020). Apoplastic proteases: powerful weapons against pathogen infection in plants. *Plant Commun.* 1:100085. 10.1016/j.xplc.2020.100085 33367249 PMC7748006

[B100] Waterhouse A. Bertoni M. Bienert S. Studer G. Tauriello G. Gumienny R. (2018). SWISS-MODEL: homology modelling of protein structures and complexes. *Nucleic Acids Res.* 46 W296–W303. 10.1093/nar/gky427 29788355 PMC6030848

[B101] Wawra S. Djamei A. Albert I. Nürnberger T. Kahmann R. Van West P. (2013). In vitro translocation experiments with RxLR-reporter fusion proteins of avr1b from *Phytophthora sojae* and AVR3a from *Phytophthora infestans* fail to demonstrate specific autonomous uptake in plant and animal cells. *Mol. Plant Microbe Interact.* 26 528–536. 10.1094/MPMI-08-12-0200-R 23547905

[B102] Wawra S. Trusch F. Matena A. Apostolakis K. Linne U. Zhukov I. (2017). The RxLR motif of the host targeting effector AVR3a of *Phytophthora infestans* is cleaved before secretion. *Plant Cell* 29 1184–1195. 10.1105/tpc.16.00552 28522546 PMC5502441

[B103] Weissbach H. Etienne F. Hoshi T. Heinemann S. H. Lowther W. T. Matthews B. (2002). Peptide methionine sulfoxide reductase: structure, mechanism of action, and biological function. *Arch. Biochem. Biophys.* 397 172–178. 10.1006/abbi.2001.2664 11795868

[B104] Williams D. R. Sternthal M. (2010). Understanding racial-ethnic disparities in health: sociological contributions. *J. Health Soc. Behav.* 51 S15–S27. 10.1177/0022146510383838 20943580 PMC3468327

[B105] Wu L. Y. Siemens J. Li S. K. Ludwig-Müller J. Gong Y. J. Zhong L. (2012). Estimating *Plasmodiophora brassicae* gene expression in lines of *B. rapa* by RT-PCR. *Sci. Hortic.* 133 1–5. 10.1016/j.scienta.2011.09.036

[B106] Yahaya N. Malinowski R. Burell M. Walker H. Petriacq P. Rolfe S. (2015). “Investigating the metabolism of clubroot-infected plants by integrating metabolomic and transcriptomic approaches,” in *Proceedings of the 2015 KSM Spring Meeting and KSM-ICWG-GSP Joint Clubroot Symposyum* (Daejeon), 1.

[B107] Yamauchi J. Takayanagi N. Komeda K. Takano Y. Okuno T. (2004). cAMP-PKA signaling regulates multiple steps of fungal infection cooperatively with Cmk1 MAP kinase in *Colletotrichum lagenarium*. *Mol. Plant Microbe Interact.* 17 1355–1365. 10.1094/MPMI.2004.17.12.1355 15597741

[B108] Yi M. Chi M. H. Khang C. H. Park S. Y. Kang S. Valent B. (2009). The ER chaperone LHS1 is involved in asexual development and rice infection by the blast fungus *Magnaporthe oryzae*. *Plant Cell* 21 681–695. 10.1105/tpc.107.055988 19252083 PMC2660637

[B109] Yu F. Wang S. Zhang W. Tang J. Wang H. Yu L. (2019). Genome-wide identification of genes encoding putative secreted E3 ubiquitin ligases and functional characterization of PbRING1 in the biotrophic protist *Plasmodiophora brassicae*. *Curr. Genet.* 65 1355–1365. 10.1007/s00294-019-00989-5 31087129

[B110] Žerovnik E. (2019). Possible mechanisms by which stefin B could regulate proteostasis and oxidative stress. *Cells* 8:70. 10.3390/cells8010070 30669344 PMC6357131

[B111] Zhang X. Liu X. Fang Z. Zhansheng L. Yang L. Zhuang M. (2016). Comparative transcriptome analysis between broccoli (*Brassica oleracea* var. *italica*) and wild cabbage (*Brassica macrocarpa* Guss.) in response to *Plasmodiophora brassicae* during different infection stages. *Front. Physiol.* 7:1929. 10.3389/fpls.2016.01929 28066482 PMC5179516

[B112] Zhou Q. Galindo-Gonzalez L. Manolii V. Hwang S. F. Strelkov S. E. (2020). Comparative transcriptome analysis of rutabaga (*Brassica napus*) cultivars indicates activation of salicylic acid and ethylene-mediated defenses in response to *Plasmodiophora brassicae*. *Int. J. Mol. Sci.* 21:8381. 10.3390/ijms21218381 33171675 PMC7664628

[B113] Zhu X. Qi T. Yang Q. He F. Tan C. Ma W. (2017). Host-induced gene silencing of the MAPKK gene PsFUZ7 confers stable resistance to wheat stripe rust. *Plant Physiol.* 175 1853–1863. 10.1104/pp.17.01223 29070517 PMC5717739

