# Supplementary figures and images for: Candidate Effectors of Plasmodiophora brassicae Pathotype 5X During Infection of Two Brassica napus Genotypes
Source: Front Microbiol. 2021 Nov 3;12:742268. doi: 10.3389/fmicb.2021.742268 (PMC8595600; doi:10.3389/fmicb.2021.742268)

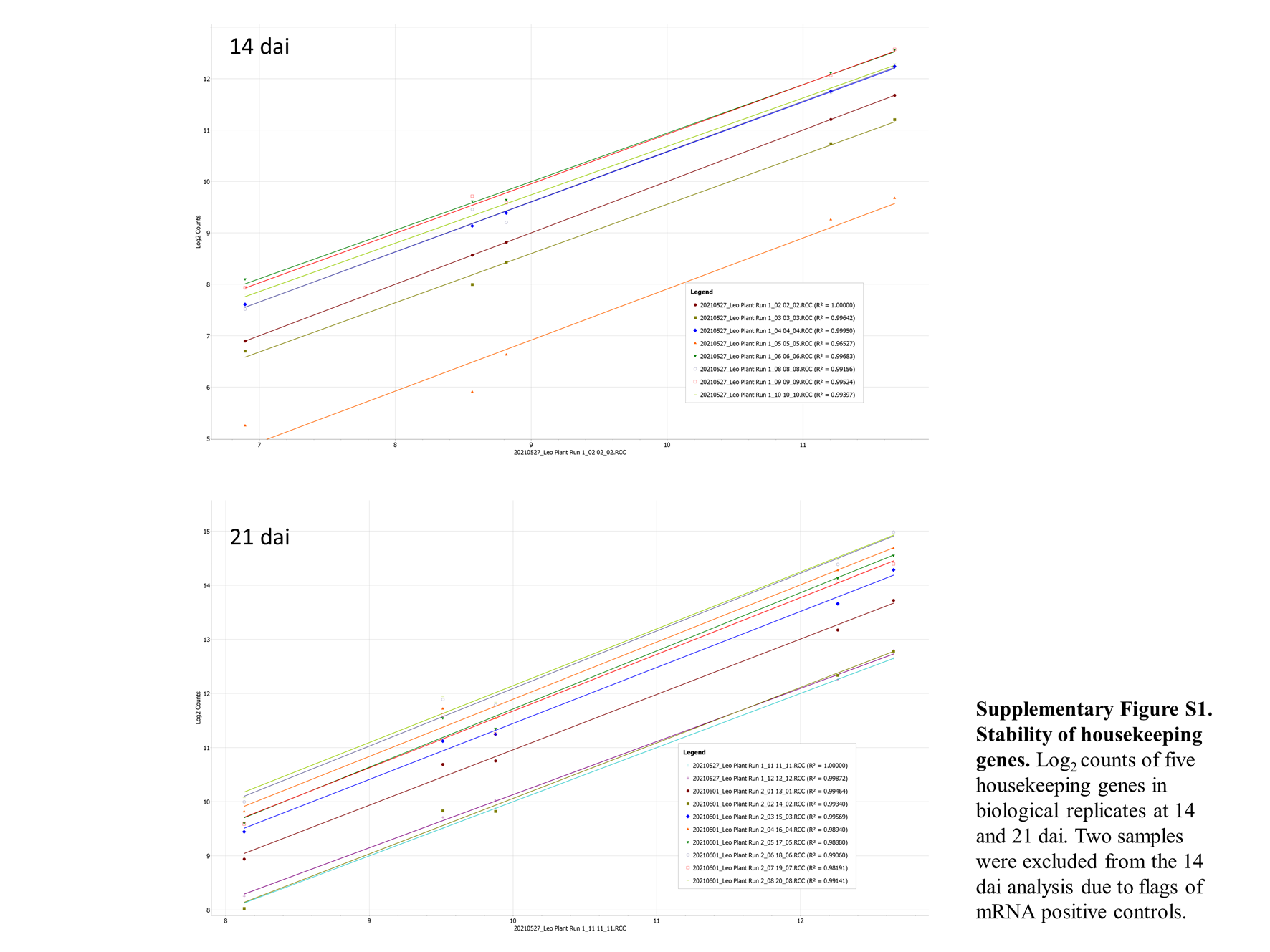

Supplement: Supplementary file 2 [file Image_1.TIF]
